# Machine Learning-Based Prediction of Stem Water Potential in Olive Orchards Using PlanetScope Imagery and Meteorological Data

**DOI:** 10.3390/s26196062

**Published:** 2026-09-24

**Authors:** Cristina Martínez-Ruedas, Samuel Yanes-Luis, Sergio L. Toral, Daniel Gutiérrez-Reina, Isabel Luisa Castillejo-González

**Affiliations:** 1 Department of Electronic and Computer Engineering, University of Cordoba, Rabanales Campus, 14071 Córdoba, Spain; z42maruc@uco.es; 2 Department of Electronic Engineering, University of Seville, Camino de Los Descubrimientos, s/n, 41009 Sevilla, Spain; syanes@us.es (S.Y.-L.); storal@us.es (S.L.T.); dgutierrezreina@us.es (D.G.-R.); 3 Department of Graphic Engineering and Geomatics, University of Cordoba, Rabanales Campus, 14071 Córdoba, Spain

**Keywords:** Extreme Gradient Boosting (XGBoost), machine learning-based irrigation management, olive orchards, PlanetScope imagery, Precision Agriculture, stem water potential (SWP), vegetation spectral indices

## Abstract

**Highlights:**

**What are the main findings?**

- PlanetScope and meteorological data enabled reliable estimation of olive water status.
- Model performance was consistent across sampling zones and growing seasons.

**What is the implication of the main finding?**

- The framework enables spatially distributed monitoring of olive water status.
- Satellite and agroclimatic data support scalable water-stress monitoring and irrigation management.

**Abstract:**

Accurate assessment of water status in woody crops is essential for optimizing irrigation management, particularly under Mediterranean conditions characterized by high spatial and temporal variability. Traditional field-based stem water potential measurements are reliable but limited for large-scale operational applications. In this study, a supervised machine learning approach based on Extreme Gradient Boosting was developed to estimate stem water potential in Mediterranean olive orchards by integrating high-resolution PlanetScope multispectral imagery with meteorological variables describing atmospheric evaporative demand. The model was trained and evaluated using a dataset comprising 1628 measurements, curated from 1856 measurements collected during 2021–2025 after temporal matching and quality-control filtering, and 128 predictive features: 44 PlanetScope-derived spectral features and 84 meteorological predictors evaluated across six temporal positions (t0–t5). Model performance was assessed using cross-validation. The model achieved a coefficient of determination of 0.84, a root mean square error of 0.27, and a mean absolute error of 0.21. Air temperature, solar radiation, and reference evapotranspiration were the most influential predictors, while spectral information captured complementary effects related to canopy structure, vegetative vigor, and accumulated physiological responses. Furthermore, the combined use of spectral indices and PlanetScope base-band reflectance values was associated with an approximately 30% lower RMSE than that reported in previous approaches.

## 1. Introduction

Efficient irrigation management in Mediterranean woody crops, such as olive groves, represents one of the most important agronomic challenges under scenarios of increasing water scarcity and climate variability [1,2,3]. In the case of olive orchards, a dominant crop in southern Europe and particularly relevant to Mediterranean agriculture, the implementation of regulated deficit irrigation strategies requires detailed knowledge of tree physiological status to optimize water use without compromising yield [4,5]. In this context, accurate assessment of crop water status is essential to optimize water use, maintain productivity, and avoid irreversible physiological impacts. Traditionally, Stem Water Potential (SWP), measured using a Scholander pressure chamber, has become the reference physiological indicator for water status of perennial trees and shrubs due to its strong theoretical basis and empirical consistency throughout the growing season [6].

SWP has established itself as a key tool for optimizing regulated deficit irrigation strategies. Multiple studies have demonstrated that the systematic use of SWP enables the precise adjustment of irrigation thresholds, avoids periods of severe water stress, and improves both productivity and water efficiency [7,8]. In olive orchards, SWP values within specific ranges allow physiological activity to be maintained without significantly reducing yield, providing an objective basis for regulated deficit irrigation scheduling [9,10,11]. In other Mediterranean fruit trees, such as almonds and pistachios, SWP-based strategies improved water use efficiency and production stability even under high evaporative demand [12]. Studies in vineyards showed that combining SWP with growth, leaf temperature, soil moisture, and meteorological data allows highly accurate modeling of water stress [13]. These results support the role of SWP in woody crop water management and the development of methods for its continuous estimation using less invasive and more scalable data sources. However, despite its reliability, SWP measurement with Scholander pressure chambers is manual, slow, and labor-intensive, limiting its use in large-scale farming systems.

Recent technologies have emerged focused on continuously monitoring the water status of crops using sensors installed directly on trees [14]. Among these approaches, xylem tension-based sensors stand out, as they allow continuous monitoring of stem water potential through micro-transducers inserted into vascular tissue [15,16]. Dendrometers measure micrometric variations in trunk or branch diameter, providing indirect estimates of plant water status from daily contraction-expansion dynamics [13,17,18]. Although both technologies represent a significant advance toward automated monitoring of water stress, they exhibit important limitations. Xylem tension-based sensors are intrusive and require direct implantation into plant tissue, while dendrometers require frequent calibration, phenological adjustments, and a detailed understanding of crop biomechanics. These limitations are aggravated by acquisition costs, the need for periodic maintenance, long-term stability issues, and the requirement for recurrent field visits, all of which hinder their large-scale operational adoption. In addition, many of these sensors show strong crop specificity, preventing their direct transfer between species and requiring the development of distinct configurations, calibration curves, and interpretative models for each case. Taken together, these constraints reinforce the need to advance toward non-invasive methodologies capable of providing robust estimates of crop water status without specialized instrumentation installed on trees.

In response to these limitations, remote sensing offers a promising alternative for assessing crop water status at spatial and temporal scales unattainable with in situ sensing alone. Satellite and aerial observations provide spectral indices sensitive to vegetation vigor, canopy structure, and plant water content [19]. Spectral indices derived from visible and near-infrared bands are widely used as indirect indicators of plant physiological status, as they capture changes in leaf pigment concentration, biomass, and canopy density, closely related to water stress dynamics [20,21,22]. The integration of these spectral indicators with ground-based physiological measurements provides a robust framework for estimating stem water potential and supporting irrigation management at the orchard scale without the need for invasive instrumentation. However, spectral indices exhibit significant limitations due to spatial resolution, environmental variability, and crop structure, making it necessary to complement them with meteorological and physiological information to improve water stress estimation. Meteorological drivers such as air temperature, vapor pressure deficit, and reference evapotranspiration regulate plant–atmosphere water exchanges and modulate crop water stress responses [23,24]. When combined with spectral indices, these variables provide complementary information that enhances the robustness and temporal stability of water status estimates [25,26]. Furthermore, the inclusion of physiological reference measurements, such as SWP, enables the calibration and validation of remote sensing-based approaches for reliable and operationally scalable assessments of crop water status [27].

Simultaneously, recent advances in data analysis techniques have encouraged the development of modeling approaches capable of integrating heterogeneous information sources and capturing complex, non-linear relationships among physiological, atmospheric, and spectral variables [28,29]. These approaches include statistical learning [30], machine learning [31], deep learning [32], and data fusion frameworks [33], which enable the joint exploitation of multivariate and multiscale datasets. Studies have applied these approaches to water stress assessment within precision agriculture and IoT-based platforms, combining sensor networks and automated irrigation systems that dynamically adjust irrigation amounts according to model outputs [34,35]. These methods have improved SWP estimation compared with traditional linear models, integrating spectral indices, meteorological variables, and canopy-derived descriptors without restrictive assumptions [36]. Moreover, advanced data analysis techniques also analyze large, multitemporal, and heterogeneous datasets to identify latent patterns, interactions, and temporal dependencies difficult to capture using classical approaches. Their flexibility facilitates adaptation to varying environmental conditions, phenological stages, and crop configurations, enhancing model robustness and spatial and temporal transferability. These capabilities support their potential for operational water management in precision agriculture.

Despite the advances described, a gap remains between research on water stress in woody crops and its application at farm scale. Many studies rely on short-term experimental campaigns, limited study areas, or technologies that, although they improve water status monitoring, require frequent field visits for sensor installation, calibration, maintenance, or validation of physiological measurements. This dependence on intensive fieldwork limits scalability and adoption in large-scale production systems. Moreover, many studies use spectral indices or environmental variables, without systematic integration with reference physiological measurements that would ensure model robustness and transferability. For olive orchards, studies combining multi-year time series, high spatial resolution satellite data, meteorological information, and direct SWP measurements to develop reliable operational predictive models remain scarce. This methodological fragmentation, together with the persistent need for field visits and limited availability of large, well-calibrated datasets, highlights the need for integrative, scalable approaches to continuous and accurate estimation of crop water status.

Several recent studies have applied remote sensing and machine learning to SWP estimation, but their constraints motivate the approach adopted here. Garofalo et al. [37] used random forest models with PlanetScope-derived vegetation indices to estimate SWP in olive orchards (R^2^ = 0.78), but the study was limited to a single site and two seasons, and accuracy dropped markedly when the model was tested on an independent year. Savchik et al. [38] predicted SWP in almond orchards from unmanned aerial vehicle (UAV) imagery, evapotranspiration, and soil moisture (R^2^ = 0.33–0.73), a design whose reliance on dedicated flights limits temporal frequency and spatial coverage at farm scale. Carrasco-Benavides et al. [39] combined UAV-based thermal imagery with artificial neural networks in cherry orchards (r = 0.83), but used a random, non-independent train-test split, which, as later confirmed by Zambrano et al. [40] for Sentinel-2 based SWP models (R^2^ falling from 0.76–0.78 to 0.59 under temporally independent validation), tends to overestimate genuine predictive performance. Recurring across these studies are single-site or short-term designs, UAV-dependent acquisition, and validation schemes vulnerable to temporal leakage. The present study addresses these limitations through a substantially larger, multi-year (2021–2025) dataset spanning different varieties and irrigation regimes, satellite-based daily revisit imagery that removes the need for dedicated aerial campaigns, and the systematic integration of meteorological predictors at multiple temporal lags integrated with spectral information.

PlanetScope offers advantages for water stress monitoring at orchard scale. The SuperDove constellation used in this study provides a nominal daily revisit frequency and 3 m spatial resolution, compared with Sentinel-2 (10 m, 5-day revisit) and Landsat (30 m, 16-day revisit), enabling closer temporal matching between satellite acquisitions and field sampling and capturing spatial variability among individual tree canopies. SuperDove imagery provides eight spectral bands, including coastal blue, green I, yellow, red edge, and near-infrared channels, supporting vegetation indices such as NDRE and more detailed characterization of canopy spectral and physiological variability. These advantages, however, come with tradeoffs: PlanetScope is a commercial, restricted-access platform, unlike the freely available Sentinel-2 or Landsat missions, which may constrain reproducibility and adoption by other research groups.

In this context, this study aims to predict SWP in Mediterranean olive orchards by integrating high-resolution PlanetScope satellite data and agroclimatic station records with reference physiological measurements obtained using a Scholander pressure chamber, which provide a low-bias assessment of plant water stress. A Gradient Tree Boosting approach, implemented through the XGBoost algorithm [41], was used to model complex non-linear relationships among meteorological, spectral, and canopy-related variables. The dataset included 1856 SWP measurements collected between 2021 and 2025, together with their corresponding meteorological variables, PlanetScope spectral band reflectance values, and vegetation indices relevant to soil–plant water status estimation. These data were consolidated into a dataset comprising 128 predictive features derived from PlanetScope spectral information and meteorological variables.

## 2. Materials and Methods

### 2.1. Study Area and Sampling Design

The study was carried out at the El Valenciano experimental farm, part of the Rural Innovation Hub [42], in Seville, Andalusia, southern Spain (37.4° N, 5.5° W, WGS-84). The area is characterized by a Mediterranean climate, with hot, dry summers and mild, wet winters, providing suitable conditions to evaluate crop water stress under semi-arid conditions. The Rural Innovation Hub is dedicated to testing and validating technologies for smart agriculture and sustainable water management. The experimental farm exhibits marked heterogeneity in management practices, water availability, and soil characteristics, supporting the evaluation of remote sensing-based and advanced data-driven models for water stress prediction in Mediterranean olive orchards.

The study area comprises 75 sampling zones distributed across rainfed conditions, conventional irrigation, and regulated deficit irrigation regimes (Figure 1a). The experimental farm includes different olive cultivars, planting configurations, and irrigation strategies. The varietal composition differed between sampling periods. During 2021–2023, the sampled areas included only Sikitita, whereas those surveyed in 2024–2025 included Arbequina, Arbosana, Sikitita, Lecciana, Sultana, and Martina. Some 2024–2025 sampling areas included rows belonging to more than one cultivar; therefore, the dataset was not structured as a cultivar-stratified experiment, and an unambiguous percentage of SWP observations could not always be assigned to each individual variety. For this reason, varietal composition is described according to the sampling campaign and spatial configuration rather than as a proportional distribution of the complete dataset. The coexistence of different plant materials under contrasting management conditions contributes to capturing a wide range of physiological and spectral variability, providing a heterogeneous dataset for the development of the proposed model. However, the sampling design was not conceived as a balanced varietal experiment, and the number of observations differed among varieties, with some cultivars being represented by a relatively limited number of samples. Accordingly, the modelling approach aimed to capture SWP variability across the heterogeneous field conditions represented in the complete dataset rather than evaluate variety-specific responses. Between 2021 and 2023, several sampling zones formed part of the CENTARIA project [43], where distinct irrigation strategies were implemented and SWP was systematically measured using a Scholander pressure chamber. The spatial distribution of these sampling zones, together with those surveyed during 2024–2025, is shown in Figure 1. Their relatively large spatial extent allowed the definition of 20 × 20 m sampling plots, spaced approximately 20 m apart (Figure 1e). During 2024 and 2025, field sampling was conducted in different sectors of the farm through intensive survey campaigns, broadening the range of water conditions captured. Because of the smaller spatial extent compared with the CENTARIA plots, these sampling areas were defined as 10 × 10 m units covering the entire analyzed area (Figure 1b–d).

The sampling period (March to October) was intentionally aligned with the olive phenological stages of highest water demand and stress sensitivity, namely flowering and fruit set, pit hardening, and oil accumulation [44] and with the period during which regulated deficit irrigation is applied in the study area, thereby capturing the full range of physiological responses relevant to irrigation management. Because this period coincides with the dry Mediterranean summer, effective precipitation between a PlanetScope acquisition and its matched SWP measurement is negligible in practice. Nevertheless, precipitation is included in the model as an explicit meteorological predictor with lags t0 to t5, so its potential effect on plant water status is represented within the model’s input space rather than acting as an unmodeled confound. In addition, although soil moisture and micrometeorological conditions were not measured with dedicated in situ sensors on each sampling date, so as to avoid the deployment of additional field instrumentation, this information is indirectly represented in the dataset: the irrigation regime of each sampling zone (rainfed, conventional, or regulated deficit irrigation) provides categorical information on its soil water management context, while the precipitation and reference evapotranspiration obtained from the SE101_IFAPA station (Section 2.2.3) characterize the atmospheric and water-balance conditions prevailing on each measurement date.

### 2.2. Data Collection

#### 2.2.1. Ground Measurements

During 2021–2025, 1856 SWP measurements were obtained using a Scholander chamber [45], which constitutes the physiological reference variable used as ground truth for training and validating the estimation models developed in this study. Of these, 1628 were retained after quality-control filtering for use in model development (see Section 2.2.4 for filtering criteria). The measurements were taken mainly during the period May–October, coinciding with conditions of maximum evaporative demand and the application of deficit irrigation, when the water response of olive trees shows greater physiological variability and an accurate estimate is most valuable.

The measurement procedure followed standardized protocols to ensure the reliability of the values obtained. In each sampling zone, fully expanded leaves were selected from the inner, shaded part of the canopy to minimize the influence of direct radiation and rapid variations in stomatal opening. Prior to extraction, the leaves were placed in aluminum bags for 10–15 min, allowing water equilibrium between the petiole and xylem, an essential step for obtaining an accurate reading of water potential [46]. After stabilization, the leaves were cut and immediately processed in the Scholander chamber, recording the SWP value in absolute megapascals (MPa). For each sampling zone and sampling date, three individual leaf water potential readings were taken with the Scholander pressure chamber and averaged into a single representative SWP value, which constitutes one measurement in the reported total of 1856. Each of these averaged, zone- and date-level SWP values was paired with a single, temporally matched PlanetScope acquisition (Section 2.2.2) and the corresponding meteorological record (Section 2.2.3), so that each row of the final dataset represents one zone-date observation with a strictly one-to-one correspondence between the SWP value and its associated remote-sensing and meteorological predictors. To ensure the reliability and comparability of these values, all three readings were taken at midday, between 13:00 and 16:00 h (local solar time), coinciding with the period of maximum canopy transpiration and atmospheric evaporative demand, thereby avoiding the additional variability that diurnal fluctuations in SWP would otherwise introduce across sampling dates and plots. Sampling dates themselves, however, were determined by the agronomic requirements derived from deficit irrigation management rather than by satellite overpass schedules, and therefore did not necessarily coincide with the PlanetScope acquisition dates. This time lag, far from being a limitation, reflects the actual conditions of crop management, where irrigation decisions are scheduled based on the physiological state of the plant and not on the availability of orbital images. Methodologically, this lack of synchrony, represented by a time lag, generates an additional physiological variable, which has been explicitly considered in the model design and allows for the evaluation of its ability to predict water status even when spectral information and field measurements are not obtained simultaneously.

#### 2.2.2. PlanetScope Data Acquisition and Vegetation Indices Estimation

High-resolution multispectral imagery from the PlanetScope nanosatellite constellation, a commercial system operated by Planet Labs Inc., was used to monitor vegetation conditions at the plot scale. Only imagery acquired by SuperDove (PSB.SD) sensors was used in this study. Although the sensors acquire imagery with a native spatial resolution ranging between 3.7 m and 4.1 m depending on orbital altitude, the data are resampled and distributed at a standardized pixel size of 3 m. Orthorectified surface reflectance products were used. SuperDove provides eight spectral bands: coastal blue (431–452 nm), blue (465–515 nm), green I (513–549 nm), green (547–583 nm), yellow (600–620 nm), red (650–680 nm), red-edge (697–713 nm), and near-infrared (845–885 nm). The near-daily revisit frequency enables continuous monitoring of crop dynamics during periods of peak water demand, reducing data gaps caused by cloud cover [47]. After project proposal approval, 16-bit orthorectified surface reflectance imagery was accessed through the Planet Education and Research Program [48]. These products are atmospherically corrected to reduce atmospheric effects and support radiometrically consistent multitemporal analyses [49].

The use of spectral indices is one of the most widely used tools in remote sensing to characterize the physiological state of vegetation [50,51]. These indices are obtained through mathematical combinations of reflectance values from different regions of the electromagnetic spectrum. The 8-band enhanced spectral configuration improves sensitivity to vegetation structure, chlorophyll content, and canopy stress. Based on these spectral bands, several vegetation indices, which are widely used to assess vegetation vigor, were computed (Table 1). The vegetation indices provide relevant information for predicting and interpreting the hydrological response of the plant in response to exogenous characteristics [52].

PlanetScope spectral bands used for index calculation. Four-band PlanetScope products include B (Blue: B02), G (Green: B04), R (Red: B06), and NIR (Near-Infrared: B08). Eight-band (SuperDove) products additionally provide CB (Coastal Blue: B01), G1 (Green I: B03), Y (Yellow: B05), and RE (Red Edge: B07).

The derived indices were grouped into structural vegetation indices (NDVI, EVI2, SAVI, OSAVI, MSAVI), chlorophyll-related indices (GNDVI, CI green), red-edge-based indices (NDRE, CI red_edge), visible-based indices (VARI), photosynthetic efficiency indices (PRI), and senescence-related indices (PSRI, SIPI). Within the structural group, soil-adjusted indices such as SAVI, OSAVI, and MSAVI were included to explicitly minimize soil background effects, particularly in woody crops with wide spacing or partial coverage, such as olive orchards. Indices whose original formulations rely on narrow or sensor-specific spectral bands, such as the PRI, PSRI, and SIPI indices, were computed using adapted or approximate formulations to ensure compatibility with SuperDove imagery. Specifically, PRI was approximated using the Green I and Green bands. Figure 2 illustrates the spatial distribution of the PSRI approximation on selected acquisition dates across the study areas, providing an example of the spatial and temporal variability in the derived indices. Corresponding false-color infrared composites are provided in Supplementary Material S1 (Figure S1).

For the study, both mean and maximum values were extracted for each original spectral band of the PlanetScope imagery as well as for all derived vegetation indices within each sampling zone. Mean values were used to characterize the overall vegetation condition by integrating intra-plot spatial heterogeneity related to management practices, soil properties, and water availability. In contrast, maximum values were considered representative of the potential vegetation response, reducing the influence of soil background and mixed pixels. The combined analysis of mean and maximum statistics enabled a robust assessment of spatial variability within sampling zones and improved the detection of localized stress patterns and temporal changes in vegetation conditions. This is consistent with related remote-sensing-based studies on crop water status: Garofalo et al. [37] characterized each sampling unit through mean and standard error statistics in a comparable PlanetScope-based olive SWP study, while Li et al. [65] showed that water deficit alters not only the mean but the upper and lower tails of the within-plot pixel-value distribution, using percentile-based features accordingly.

#### 2.2.3. Meteorological Data

The meteorological data used in this study were obtained from the SE101_IFAPA Centro Las Torres-Tomejil agroclimatic station [66], located approximately 10 km from the El Valenciano experimental farm. This station-to-farm distance (~10 km) is consistent with the typical spacing of public agroclimatic networks operating in the region, such as IFAPA and SIAR, which are designed on the assumption that standard agroclimatic variables (air temperature, solar radiation, and reference evapotranspiration) are spatially representative over homogeneous agricultural plains at this scale. The area between the SE101_IFAPA station and the El Valenciano farm corresponds to a flat, agriculturally homogeneous landscape, without significant elevation changes, orographic features, or land-cover discontinuities. The station is operated and periodically calibrated by public authorities, ensuring data quality and reliability. Its use allows methodological standardization and guarantees the reproducibility of the results, without the need to deploy additional field instrumentation or to introduce technical dependencies on the participating farms [67].

From this station, the meteorological variables effectively used in the analysis were collected, including daily mean, maximum and minimum air temperature (with their corresponding time of occurrence), daily mean, maximum and minimum relative humidity (with their corresponding time of occurrence), global solar radiation, cumulative and effective precipitation, and reference evapotranspiration (ET_0_) (see Table 2).

Each time series was temporally synchronized with the field sampling schedule and the satellite acquisition dates. For each meteorological variable, three daily metrics were calculated: the maximum value, the minimum value, and the mean of all values throughout the day. This information is particularly relevant for variables with high intra-daily variability, such as temperature and relative humidity, for which extreme values may be more representative of crop stress conditions.

For the construction of the dataset, it was necessary to account for the temporal dependencies between meteorological variables from previous days and the measurement at the current time t. Accordingly, for each SWP observation, six meteorological records were included for each variable, temporally shifted by one-day intervals (lags), starting from the day of the SWP measurement (lag 0) until 5 days before. Thus, for each prediction, up to a maximum of six lagged values were considered ($M_{t},\;M_{t-1},\;\ldots ,\;M_{t-5}$).

The integration of these open-access meteorological data enhances the practical applicability of the developed model, enabling its use in farms that do not have on-site weather stations and facilitating its transferability to other agricultural environments with similar conditions.

#### 2.2.4. Dataset Curation and Description

The dataset used in this study was constructed through the integration of spectral, meteorological, and physiological information described in Section 2.2.1, Section 2.2.2 and Section 2.2.3, resulting in a dataset that combines observations from different sources for each study area. In total, 1856 SWP values were obtained, corresponding to the 1856 field measurements acquired using a Scholander pressure chamber. These measurements essentially constitute the target variable for the prediction task. The final dataset comprised 129 variables in total, including SWP and 128 predictive features. Of these predictors, 44 were derived from PlanetScope imagery, including 16 features corresponding to the mean and maximum reflectance values of the eight spectral bands and 28 features corresponding to the mean and maximum values of 14 vegetation and spectral indices. The remaining 84 predictors were derived from meteorological information and corresponded to 14 variables evaluated at six temporal positions (t0–t5). Thus, the final predictor set integrated 44 spectral and 84 meteorological features, while SWP was used exclusively as the response variable. Following the temporal matching between each SWP measurement and its closest PlanetScope acquisition (Section 2.2.2), a quality-control step was applied to remove records with incomplete predictor data—primarily rows lacking red-edge-derived indices (NDRE, CI red-edge), available only for SuperDove 8-band acquisitions (84 of the 89 dates analyzed)—together with duplicate records. This filtering reduced the dataset from 1856 to a final curated set of 1628 complete SWP records, which constitutes the dataset used for feature engineering, model training, and validation throughout the remainder of this study. Figure 3 shows the overall distribution of SWP values in the dataset. The data are expressed as absolute values in MPa (since SWP is always negative) and span a range from 0.80 to 4.5 MPa, where higher SWP values indicate greater levels of crop water stress. It can be observed that the frequency of SWP values above 2 MPa progressively decreases, reflecting the tendency to minimize water stress through deficit irrigation strategies. This aspect is not trivial, as the prediction of high SWP values is particularly important due to the increased risk posed to the crop under such conditions. Consequently, when applying data-driven estimation techniques, it is essential to consider dataset stratification strategies to mitigate the imbalance of observations in the upper range of SWP values.

Figure 4 shows the distribution and frequency of SWP values as a function of the ordinal week of the year. This distribution indicates that the data were collected from early March to late October. The frequency of SWP datapoints, grouped by ordinal week, reveals the presence of sample clusters associated with the different farms. A temporal correlation between SWP and the ordinal week of the year can be observed, driven by the meteorological variables characteristic of each month of the growing season.

The variability of the data as a function of the time of year introduces two levels of difficulty in SWP estimation. During the early months of the season (March–May), milder temperatures and a less pronounced lack of precipitation make SWP easier to predict, as it remains relatively stable. It is during the second and third quarters of the year that water stress can increase sharply, depending on specific irrigation conditions, temperature, humidity, and other environmental factors.

Regarding the aggregation of PlanetScope data into the dataset—including both the original spectral bands and the derived spectral indices—it must be considered that temporal mismatches may occur between PlanetScope acquisitions and SWP measurements. This misalignment arises from differences between the timing of SWP measurements, which are conducted by technicians according to farm operations, and the satellite overpass frequency of PlanetScope. To mitigate the need to restrict the dataset to SWP measurements acquired only on days with PlanetScope data, the closest available PlanetScope observation within a ±96 h temporal window was selected. It is reasonable to assume that, due to the continuous nature of crop dehydration processes, PlanetScope-derived index values remain valid over a short temporal window, provided they are acquired at a similar time of day. In this context, each SWP observation was assigned a one-to-one correspondence with a PlanetScope data record, with a maximum absolute temporal lag of 96 h. The sensitivity of model prediction error to this temporal lag is examined in Section 3.3.4.

Figure 5 shows the histogram of the data as a function of the temporal difference between each SWP measurement and its corresponding PlanetScope observation. It can be observed that approximately half of the PlanetScope data were acquired on the same day as the SWP measurement. This represents an advantage of the PlanetScope constellation, which provides an almost daily acquisition frequency with a maximum temporal offset of less than four days. The remaining samples with lags greater or smaller than 24 h are, in most cases, associated with PlanetScope data unavailability due to missing images or cloud cover over the study area.

From the aggregation of PlanetScope and meteorological variables, clear correlation relationships between variables can be derived and visualized. In Figure 6, two variables are shown that allow the identification of noisy linear and non-linear relationships with SWP. The large volume of available data enables the development of a reliable predictive model for estimating crop water stress.

### 2.3. Data-Driven Model

The prediction of water stress can be addressed using different Machine Learning or Deep Learning algorithms. In previous studies, approaches based on Random Forest Regression or Support Vector Regression [37] have been commonly adopted. In general, the application of these algorithms has relied on a black-box approach, in which models are primarily compared based on their performance metrics. In the present study, olive variety was not included as an exogenous predictor. Instead, the modelling framework was designed to estimate SWP across the heterogeneous field conditions represented in the complete dataset, rather than to develop or evaluate variety-specific models. In this section, the proposed methodology is described, which is based on Gradient Tree Boosting using the XGBoost algorithm [41].

### 2.4. XGBoost for the Estimation of Agronomic Variables

Decision trees are supervised models that approximate nonlinear relationships through successive partitions of the feature space. At each split, a threshold is selected on a predictor variable with the aim of reducing the dispersion of the response variable in the resulting nodes. This hierarchical construction allows interactions among predictors to be captured without imposing parametric assumptions on the data distribution. However, an individual decision tree is often unstable and sensitive to noise, leading to a high risk of overfitting in complex regression problems.

To mitigate this risk, the boosting paradigm was introduced, which consists of additively combining multiple decision trees trained in a sequential manner. Each subsequent tree is fitted using the residual errors of the model accumulated up to that point, thereby progressively improving predictive performance. This procedure can be interpreted as a functional optimization process that minimizes a loss function through the iterative incorporation of weak learners. In this way, bias and variance are balanced, yielding more robust models without the need to excessively increase tree depth (Figure 7).

XGBoost (Extreme Gradient Boosting) implements this approach through a formulation that improves both computational efficiency and overfitting control. The objective function includes an explicit regularization term that penalizes tree complexity, preventing the generation of unnecessary splits and enhancing generalization capability. Its training algorithm relies on a second-order gradient approximation, computing not only the gradient but also Hessian information to estimate the expected gain of each split. This treatment enables the selection of partitions that optimize error reduction in a more stable and effective manner. In addition, the computational design of XGBoost incorporates parallelization, efficient threshold search strategies, and optimized handling of sparse data, enabling its application in scenarios involving large datasets and high dimensionality.

These properties are particularly well suited for the estimation of water stress in olive orchards using meteorological variables and spectral indices derived from remote sensing. In this domain, the relationships between predictors and the response variable are highly nonlinear and may involve interactions that depend on time and on the physiological state of the crop. XGBoost is able to capture such interactions without requiring the manual specification of complex transformations, and it provides feature importance measures that allow an assessment of which variables contribute most to the prediction. This capability supports a degree of model interpretability, facilitates the identification of relevant spectral or meteorological indicators, and can inform the design of monitoring or agricultural intervention strategies. Moreover, its computational efficiency enables the execution of comparative analyses, cross-validation procedures, and interactions with feature selection techniques within reasonable time frames.

## 3. Experiments and Results

The following section presents the results obtained from the application of XGBoost for SWP prediction using the proposed dataset. First, the results of the hyperparameter optimization study are reported. Subsequently, an ablation study is presented to analyze the individual contribution of each feature family to the final model performance. Finally, a comparison with algorithms and methods reported in previous studies is carried out to validate the robustness and relevance of the obtained results (https://gitlab.ratatosk.cc/syanes/estimacion-de-estres-hidrico-en-olivos/DATA (accessed on 21 September 2026)).

### 3.1. Dataset Preparation for Training and Validation

For all training procedures, the dataset was split into 80% for training and 20% for validation. Due to the uneven distribution of the data across SWP values, a stratified split was adopted to ensure that both the training and validation sets contained a proportional representation of each SWP range.

For this purpose, the SWP values were divided into 10 intervals spanning from the minimum value (0.797 MPa) to the maximum value (4.5 MPa). Within each interval, samples were assigned to the training and validation sets according to the 80/20 ratio. As a result, both datasets exhibit a similar distribution across the full range of available SWP values, thereby preventing the overrepresentation of specific measurements and serving as a mechanism to control bias.

For both model training and the ablation studies, cross-validation was applied to obtain the optimal model configuration. The cross-validation (CV) technique consists of dividing the training set into equally sized subsets. For each subset (fold), the model is trained using only the data outside that fold. In this study, five folds were used for all experiments. Final performance was computed as the average performance obtained across the models trained while excluding each fold in turn. This approach yields results with reduced bias with respect to the data used for training and can be employed as a criterion for hyperparameter or model architecture selection. For comparisons among different algorithms, however, predictions on the validation dataset were used, as these data were never included in any fold during the training process.

It should be noted that SWP measurements are not independent across observations: samples originate from a limited number of sampling zones that are repeatedly monitored across multiple dates and growing seasons, and the associated PlanetScope and meteorological predictors vary smoothly over time. Consequently, a purely random row-level split, as employed above, may place near-duplicate observations (same plot, nearby dates) in both the training and validation partitions. To assess the extent to which the reported performance depends on this row-level randomization, two additional validation schemes, grouped by sampling node and by growing season, were implemented and are reported in Section 3.3.3.

### 3.2. Performance Metrics

The following metrics are described to assess the optimality of the models and the baseline approaches:

Root Mean Squared Error (RMSE): This metric quantifies the average magnitude of prediction errors while penalizing large residuals more heavily because errors are squared before averaging. Consequently, RMSE is relatively sensitive to outliers and large prediction errors.

$$RMSE\left(y,\hat{y}\right)=\sqrt{\frac{1}{N}\sum_{i=0}^{N}{\left(y-\hat{y}\right)}^{2}}$$

Mean Absolute Error (MAE): This metric represents the mean absolute difference between predicted and observed values. Because errors contribute linearly rather than being squared, MAE is less sensitive to outliers than RMSE.

$$MAE\left(y,\hat{y}\right)=\frac{1}{N}\sum_{i=0}^{N}\left|y-\hat{y}\right|$$

Determination coefficient ($R^{2}$): It represents the correlation between the predicted and observed values. This metric measures how much of the variance in the data is explained by the model, within a range of [0, 1], where 1 corresponds to a perfect model. It is useful for comparing models with one another, provided that the number of data points remains constant, since its denominator increases proportionally with the sample size.

$$R^{2}\left(y,\hat{y}\right)=\frac{1-\sum_{i=0}^{N}{\left(y-\hat{y}\right)}^{2}}{\sum_{i=0}^{N}{\left(y-\bar{y}\right)}^{2}}$$

### 3.3. XGBoost Hyperparametrization

In any application of ML/DL algorithms, the effect of hyperparameters on final performance should be systematically analyzed. Given the large number of training and validation parameters involved in algorithms such as XGBoost, it is necessary to investigate which parameter values can maximize prediction optimality. To obtain an efficient model, the search was focused on identifying the optimal values of the hyperparameters listed in Table 3. These hyperparameters are those that are commonly reported to have the greatest influence on regressor performance [69].

For hyperparameter optimization, a Tree-structured Parzen Estimator (TPE)-based search algorithm [70] was employed using all available feature types. Unlike grid or random search, TPE is a sequential model-based optimization method: rather than modeling the objective metric directly as a function of the hyperparameters, it models the inverse relationship, that is, the density of hyperparameter values conditioned on performance. At each iteration, previously evaluated configurations are split, according to a quantile threshold on the cross-validated loss, into a ‘good’ and a ‘poor’ subset, and a kernel density estimate is fitted separately to each. The next candidate configuration is then drawn from the region where the ratio between the good-configuration density and the poor-configuration density is highest, which has been shown to be asymptotically equivalent to maximizing the expected improvement used in classical Bayesian optimization, while remaining computationally cheaper and better suited to mixed discrete-continuous, and potentially conditional, hyperparameter spaces such as the one considered here (Table 3). This progressively concentrates the search in promising regions instead of sampling the space uniformly, which is particularly advantageous given the six-dimensional, mixed-type hyperparameter space explored in this study.

The optimized configuration in Table 4 reflects a coherent regularization strategy rather than an arbitrary combination of values. The low learning rate (0.013) combined with a large number of estimators (619) favors a slow, incremental boosting regime in which each tree contributes only a small correction to the ensemble, reducing the risk of overfitting to noise in the 1628-sample curated dataset while still allowing the cumulative model to capture complex, non-linear relationships. The relatively high maximum depth (17) indicates that individual trees are allowed to model detailed feature interactions, which is offset by a moderate column subsampling ratio (0.507), exposing each tree to roughly half of the 128 predictors and thereby decorrelating the trees within the ensemble despite their depth. The near-unity row subsampling ratio (0.937) suggests that, unlike the feature dimension, limiting the number of training rows per tree offered little additional benefit, likely because the dataset does not contain a large fraction of redundant or highly repetitive observations. Finally, the moderate minimum child weight (4.840) discourages splits supported by very few, potentially noisy observations. Taken together, these values are consistent with a search for a highly expressive but well-regularized model, and help explain the measurable, if moderate, improvement over the default configuration reported in Table 5 (RMSE reduced by approximately 7%, R^2^ increased by approximately 3%): rather than reflecting a fundamentally different model, TPE identifies a more favorable bias-variance trade-off within the same XGBoost framework.

The study was conducted using 100 iterations of the TPE algorithm. The final parameter values obtained from this process are reported in Table 3.

The comparison shows that TPE-based optimization consistently improves generalization metrics with respect to the default configuration (Table 4). Hyperparameter optimization using TPE systematically enhances performance: RMSE is reduced by approximately 7%, MAE by a similar margin, and R^2^ increases by around 3%. These differences indicate that hyperparameter tuning provides measurable benefits over default parameter settings for SWP estimation, resulting in lower average prediction error and a higher proportion of explained variance (Table 5). Finally, Figure 8 illustrates the predicted values in comparison with the observed values for the test set.

#### 3.3.1. Feature Importance Analysis

XGBoost provides several metrics to quantify the importance of each feature in the model. Two of the most commonly used are Gain and Weight. Both are derived from the structure of the trees generated, but they reflect different aspects of each predictor’s contribution.

The analysis of feature importance in XGBoost using the Gain and Weight metrics provides complementary insights into the role of each predictor in SWP estimation using meteorological and spectral data. Gain quantifies the average reduction in the loss function associated with the use of a given feature in internal tree splits. It therefore identifies variables whose marginal contribution to error reduction is high, even if they are used infrequently. Weight, in contrast, measures the frequency with which a feature is used in splits across all trees in the model, providing information on its structural recurrence within the decision process.

These metrics make it possible to assess the informative capacity of each type of variable. A high Gain value for certain PlanetScope bands or indices would suggest that they contain information sensitive to physiological changes related to water stress, even if their contribution appears in specific and non-recurrent splits. Conversely, a high Weight value for meteorological predictors would indicate that these variables are systematically used by the model, reflecting their consistent relevance under different environmental conditions.

After training XGBoost with the optimal set of parameters, the importance of each feature can be examined in terms of its Gain (Figure 9) and Weight (Figure 10). The results reveal that minimum air temperatures dominate the Gain importance in the resulting estimator. This finding suggests that temperature variability contains highly discriminative information regarding the water status of olive trees. From a physiological perspective, this implies that temperature changes are strongly associated with plant or soil water behavior, possibly through their relationship with processes such as transpiration, evaporative demand, and stomatal regulation.

On the other hand, when analyzing the Weight importance of the resulting model, vegetation indices—and particularly the maximum values of the base spectral bands—are found to dominate. This indicates that the XGBoost model relies more frequently on these features across the trees to perform splits. Although the magnitude of improvement at each split may be moderate, their repeated use suggests that they provide consistent and useful information for discriminating SWP levels across different data subsets.

This recurrence implies that extreme variations (maximum values) in these bands, as well as the behavior of the PRI, contain detectable and persistent signals related to crop water status, which are associated with plant physiological responses such as changes in reflectance linked to chlorophyll content, photosynthetic efficiency, or stomatal regulation.

In the following experiment, feature importance was analyzed in relation to model accuracy, using RMSE as the reference metric. To assess the practical relevance of the features in terms of their contribution to the final error, an ablation study was conducted considering five feature set configurations, in addition to a baseline case including all features. These feature sets were defined as follows: (i) meteorological data only, including all temporal lags; (ii) PlanetScope data only, comprising both base spectral bands and derived indices; (iii) meteorological data with temporal lags combined with PlanetScope vegetation indices; (iv) meteorological data without lags combined with all PlanetScope data; and (v) complete meteorological data combined with PlanetScope base spectral bands only.

Figure 10 shows the RMSE obtained when training XGBoost on the training dataset and evaluating it on the test dataset. The results of the ablation study (Figure 11 and Table 6) indicate that the progressive removal of feature groups leads to a systematic increase in error, reflected in both RMSE and MAE, together with a decrease in the coefficient of determination (R^2^). The model including all variables achieves the best overall performance (RMSE = 0.282), suggesting that the joint combination of meteorological predictors, temporal lags, and spectral variables captures complementary information that is relevant for SWP estimation.

The exclusion of specific feature groups produces differentiated effects. The feature set composed of meteorological variables with temporal lags and base spectral bands maintains performance close to the full model (RMSE = 0.295), indicating that base bands provide relevant information and that vegetation indices are not critical when meteorological data are exploited with temporal memory. Similarly, removing meteorological lags results in a moderate performance penalty (RMSE = 0.298), highlighting that the inclusion of temporal dependencies, while beneficial, is not the primary determining factor of predictive capability.

The feature set combining meteorological variables with lags and PlanetScope vegetation indices leads to a more pronounced degradation in performance (RMSE = 0.318). This increase suggests that raw spectral bands contain more direct or stable information for inferring water stress than derived indices, at least under the experimental conditions considered. Finally, the model excluding meteorological data exhibits the largest loss in performance (RMSE = 0.377), revealing that atmospheric variables constitute the dominant component in SWP prediction, whereas spectral features alone are insufficient to adequately characterize water stress variability.

These empirical results are consistent with the interpretation derived from the previous Gain and Weight analyses: meteorological variables—particularly temperature and variables related to evaporative demand—produce substantial reductions in the loss function (high Gain), while base bands and indices such as PRI appear recurrently across the trees (high Weight), providing complementary but insufficient information on their own. Overall, the ablation study empirically confirms the practical relevance of both feature groups and indicates that the integration of atmospheric predictors with satellite base spectral bands constitutes an efficient combination for estimating SWP using XGBoost models.

#### 3.3.2. Comparison with Other Algorithms

Multiple linear regression (MLR) constitutes the simplest baseline model in the comparison. It is based on the assumption that the target variable can be expressed as a linear combination of the predictors, with coefficients estimated by least squares. Its low performance (RMSE = 0.49; R^2^ = 0.58) indicates that the relationship between meteorological and spectral predictors and SWP is strongly nonlinear and non-additive, rendering this model insufficient to represent the physiological dynamics of the crop.

Random Forest (RF) employs an ensemble of decision trees trained using bagging, that is, bootstrap subsets of the data and random subsets of features at each split. This strategy reduces estimator variance by averaging multiple uncorrelated predictors. Although it substantially improves upon linear regression (RMSE = 0.38; R^2^ = 0.78), its ability to capture complex interactions appears limited when compared to boosting-based methods, in agreement with previous studies [37].

Gaussian Processes (GP) with an RBF kernel constitute a nonparametric probabilistic approach capable of modeling nonlinear relationships through covariance functions. Each prediction is accompanied by an uncertainty estimate, which is valuable in agronomic applications. However, their performance (RMSE = 0.34; R^2^ = 0.75) reveals difficulties in generalizing under high-dimensional settings and potential collinearity among satellite and meteorological predictors, in addition to the high computational cost involved.

Support Vector Regression (SVR) extends SVM to the regression setting by constructing a prediction function that minimizes an error margin defined by the epsilon-insensitive loss. With appropriate kernels, it can capture nonlinear relationships. The results (RMSE = 0.30; R^2^ = 0.81) indicate that the method models the problem reasonably well, although it underperforms relative to XGBoost, possibly due to limitations in handling nonlinear interactions among multiple heterogeneous features.

The implemented dense neural network employs several hidden layers with 128, 256, and 64 neurons, respectively, providing an architecture capable of approximating complex functions. However, the achieved performance (RMSE = 0.37; R^2^ = 0.72) indicates that, even after optimization, the model fails to stably capture the relationship between predictors and SWP, likely due to the limited size of the dataset.

Finally, XGBoost combines multiple trees trained sequentially to correct residual errors through an objective function that includes regularization terms, facilitating overfitting control and training efficiency. Its second-order gradient formulation and ability to handle heterogeneous data explain its superior performance (RMSE = 0.27; R^2^ = 0.84; MAE = 0.21). This result supports the conclusion that interactions among meteorological variables, temporal variability, and spectral information can be effectively modeled using regularized boosting. Compared with linear methods or dense neural networks, XGBoost offers clear advantages in terms of stability, relative interpretability, and predictive performance, consolidating its suitability for estimating olive water stress from remote sensing and meteorological data. Table 7 summarizes the different algorithms considered in the comparison.

#### 3.3.3. Assessment of Spatial and Temporal Leakage in the Validation Protocol

The stratified 80/20 split and the 5-fold cross-validation scheme used in the previous sections operate at the observation (row) level. Because SWP measurements are spatially clustered in a limited number of sampling zones and temporally clustered within growing seasons, this procedure does not guarantee that samples from the same plot or the same season are kept exclusively within either the training or the validation set. To evaluate whether the reported performance is robust to this potential source of leakage, two additional validation experiments were carried out using the same optimized XGBoost configuration (Table 4) and the complete set of 129 variables: (i) a node-grouped holdout, in which sampling zones were assigned exclusively to either the training or the validation partition; and (ii) a leave-one-season-out holdout, in which the model was trained on the 2021–2023 campaigns and validated exclusively on the independent 2024–2025 campaigns.

Table 8 summarizes the results, alongside the original random stratified 80/20 split for reference. The node-grouped holdout achieved RMSE = 0.294, MAE = 0.218, and R^2^ = 0.837, only marginally worse than the original random split (RMSE = 0.282, R^2^ = 0.843), indicating that spatial proximity between training and validation samples does not substantially inflate the reported performance. The leave-one-season-out holdout showed a larger, though still moderate, degradation (RMSE = 0.320, MAE = 0.238, R^2^ = 0.817). This comparatively larger drop is consistent with the slowly evolving nature of meteorological forcing and vegetation status within a growing season: consecutive-day samples from the same campaign share mutually reinforcing temporal information that remains available to the model under random and node-grouped splits but is entirely absent when an independent season is held out. Taken together, these results confirm that the predictive performance reported throughout this study is not primarily an artifact of near-duplicate samples shared between training and validation sets, and that the model retains good generalization capability (R^2^ ≥ 0.75) even under stricter, leakage-free validation protocols.

#### 3.3.4. Sensitivity of Prediction Error to the Temporal Lag Between SWP Measurements and PlanetScope Acquisitions

As described in Section 2.2.4, each SWP measurement was paired with the closest available PlanetScope acquisition within a maximum absolute temporal lag of 96 h, since sampling dates were determined by agronomic requirements rather than by satellite overpass schedules (Figure 5). To assess whether this temporal mismatch introduces a systematic source of error, the mean prediction error on the validation set was computed within the same 24 h lag bins used in Figure 5, where a negative lag indicates that the PlanetScope acquisition precedes the SWP measurement (Figure 12).

The large majority of validation samples (277 of 326, approximately 85%) fall within one day of zero lag, consistent with the overall distribution reported in Figure 5. Mean error across all lag bins ranges between 0.06 and 0.27 MPa, with no monotonic increase as the absolute lag grows; the bins with the largest absolute lag (72–96 h and −96 to −72 h) in fact show among the lowest mean errors, although these bins contain few samples (n = 3–6) and should be interpreted with caution given their limited statistical support. The two bins with the highest sample counts and therefore the most statistically reliable estimates (−24–0 h, n = 214, and 0–24 h, n = 63) show mean errors of 0.263 and 0.217 MPa, respectively, in line with the overall validation error reported in Table 6. These results indicate that prediction error does not degrade systematically as a function of the temporal mismatch between ground and satellite observations within the adopted ±96 h window, supporting the validity of the temporal matching criterion used to construct the dataset.

## 4. Discussion

The results of this study reinforce that estimating water status in woody crops cannot rely solely on spectral information and requires explicit integration of atmospheric variables such as evapotranspiration. In this regard, our findings are consistent with those reported by [40], who demonstrated that meteorological predictors such as air temperature and reference evapotranspiration (ETo) explain a larger fraction of the daily variability of stem water potential than spectral indices considered in isolation.

The direct comparison with the study by [37] is particularly relevant, given the high degree of methodological similarity and the comparable agronomic context. In that work, conducted in Mediterranean olive orchards and based on high spatial resolution PlanetScope imagery, it was shown that machine learning models achieve their best performance when combining base spectral bands and vegetation indices (VIs), with multimodal approaches clearly outperforming purely spectral ones. However, [37] also reported a significant loss of predictive capability when transferring the models to independent seasons, highlighting the sensitivity of optical indices to interannual variability in atmospheric and phenological conditions.

With regard to the final accuracy of the model, the present results indicate that the use of a substantially larger dataset, together with careful data preparation and the XGBoost algorithm, was associated with improved predictive performance compared with previous work [37]. While that study used only 192 SWP samples for training and validation, the dataset used in the present study contains almost ten times more samples. The combination of the larger number of data points, careful data preparation, and the XGBoost algorithm resulted in an RMSE approximately 30% lower than that reported in the previous work. Moreover, beyond the merit figures, a cross-validation approach has been conducted to present the accuracy scores without data bias.

An important consideration regarding model generalizability is the heterogeneous composition of the dataset. Although the field observations encompass different irrigation regimes and several olive varieties, the sampling design was not balanced across these groups, and some varieties were represented by a relatively limited number of observations. Consequently, variety-specific or irrigation-regime-specific performance metrics were not considered sufficiently robust for separate interpretation. The results should therefore be understood as evidence of model performance across the heterogeneous conditions represented in the complete dataset, rather than as demonstrating equivalent predictive performance for each individual variety or management regime. Further studies using larger and more balanced datasets will be required to assess model transferability across cultivars and irrigation strategies in greater detail.

Within this framework, the results presented here confirm that specific PlanetScope-derived indices play a relevant role in water stress prediction [19]. Indices associated with canopy vigor and structure, such as PRI, VARI, PSRI, and EVI2, show a consistent contribution, especially under conditions of high spatial heterogeneity within the crop. These indices capture changes in leaf density and photosynthetic activity that reflect integrated responses to water stress, rather than the short-term daily dynamics of stem water potential. Likewise, indices more sensitive to water content and chlorophyll concentration, such as combinations based on green and near-infrared (NIR) bands, exhibit a higher relative importance during phenological stages of maximum vegetative activity, in agreement with the findings of [37].

Nevertheless, both in our study and in the reference work, the reflectance values of PlanetScope base bands, particularly the NIR and red bands, provide complementary and highly valuable information when directly integrated into the models, avoiding the information loss inherent to the formulation of normalized indices. This observation is consistent with recent studies in olive [10], almond [16], and pistachio orchards [12], where raw spectral bands combined with non-linear learning algorithms improve model stability against changes in canopy structure and illumination conditions.

From an atmospheric perspective, our results confirm that air temperature, solar radiation, and ETo are the most influential predictors, in line not only with [40] but also with studies conducted in vineyards, stone fruit orchards, and citrus crops [71]. The dominance of these variables highlights that stem water potential largely represents a direct response to instantaneous evaporative demand, while spectral information acts as a modulating factor that incorporates effects related to canopy structure, vigor, and accumulated physiological status.

A key aspect emerging from the comparison with the analyzed studies is that the exclusive use of meteorological variables may introduce biases when the agrometeorological station is not fully representative of the crop’s microclimatic conditions. In the present study, the SE101_IFAPA station is located approximately 10 km from the experimental farm; however, this separation lies within the standard representativeness range assumed for public agroclimatic networks over flat, homogeneous agricultural terrain, and no orographic or land-cover discontinuities are present between the two sites that would be expected to generate a substantial microclimatic gradient. In this context, the integration of PlanetScope-derived indices enables the capture of the spatial response of the canopy to atmospheric forcing, thereby reducing the uncertainty associated with the spatial separation between meteorological sensors and the agricultural plot. This synergy explains the superior performance of multimodal models compared to those based on a single source of information. This complementarity is consistent with the ablation results reported in Section 3 (Table 6), where the model excluding meteorological data shows the largest performance loss (RMSE = 0.377) and the full multimodal model achieves the best performance (RMSE = 0.282), indicating that the spectral and meteorological sources of information are complementary and that the spectral component may help account for residual spatial variability not fully represented by the meteorological input.

Overall, the evidence suggests that the combination of multiple PlanetScope spectral indices, base band reflectance values, and key meteorological variables constitutes a rich and complementary source of information for the accurate estimation of crop water status. Compared to approaches based exclusively on vegetation indices, the proposed integration allows the simultaneous capture of atmospheric demand, canopy structure, and internal plant physiological processes. These results further support the role of multimodal models as advanced tools for water stress monitoring and decision-making in precision irrigation systems, particularly in Mediterranean woody crops characterized by high spatial and temporal heterogeneity.

Despite these results, several limitations warrant consideration. First, phenological stage was not incorporated into the model as an explicit predictor. As detailed in Section 2.1, the sampling campaign was deliberately concentrated within the olive phenological stages of highest water demand and stress sensitivity (flowering and fruit set, pit hardening, and oil accumulation), for which SWP is expected to be most informative and for which the relationship between spectral indices and water status is expected to be most stable. Nonetheless, because seasonal changes in PlanetScope-derived spectral indices are inherently coupled with phenological progression, some residual confounding between phenology-driven and stress-driven spectral variation cannot be fully ruled out even within this window, and the dataset does not cover the full range of olive phenological stages. Second, although the SE101_IFAPA agroclimatic station lies within the range of representativeness assumed for regional networks, as discussed above, meteorological variables were nonetheless obtained from a station located 10 km from the experimental plots rather than from in situ sensors, which may introduce residual microclimatic discrepancies not captured by the model.

Individual irrigation events within the ±96 h matching window were not logged, as scheduling is grower-controlled; more generally, this window itself reflects a structural characteristic of the dataset, since sampling dates were dictated by agronomic requirements rather than by satellite overpass schedules. This is unlikely to affect the results: 91% of samples fall within [−96, +24] hours of the SWP measurement, the lag-based error analysis (Section 3.3.4, Figure 12) shows no systematic degradation in prediction accuracy at larger lags, and the node-grouped/leave-one-season-out validation (Section 3.3.3) confirms stable performance across independent validation schemes. Logging individual irrigation events and tightening field-sampling scheduling around satellite acquisitions are both identified as directions for future work.

With regard to feature redundancy, several meteorological predictors, most notably reference evapotranspiration (ETo), are themselves derived from other included variables such as temperature and solar radiation, introducing a degree of multicollinearity (Figure 13; Pearson r up to 0.84 between ETo and temperature). This is not expected to compromise XGBoost’s predictive performance, since tree-based splits handle correlated inputs without the instability that affects linear models; it does mean, however, that the Gain/Weight attribution between correlated variables can be somewhat arbitrary at the margin. As this study prioritizes operational prediction over disentangled causal attribution, a collinearity-aware interpretability analysis is left for future work. The ablation study (Table 6) and the node-grouped/leave-one-season-out validation (Section 3.3.3) further indicate that this collinearity does not destabilize the model, showing consistent, physically coherent performance rather than the instability expected from spurious splits.

Finally, the proposed explanatory framework implicitly assumes that soil moisture is not a limiting factor for transpiration. The dataset does not include direct soil-moisture or root-zone measurements, and this boundary condition is therefore not explicitly modeled. The wide range of SWP values recorded in this study (0.80–4.5 MPa) indicates that the sampled trees experienced conditions from well-watered to severely water-stressed, supporting the general validity of the proposed framework across this range; incorporating direct soil-moisture measurements to explicitly delimit its applicability under conditions of severe soil-water limitation is left for future work.

## 5. Conclusions and Future Works

The results confirm that reliable estimation of water status in woody crops cannot rely solely on spectral information but requires explicit integration of atmospheric variables characterizing evaporative demand. In particular, air temperature, solar radiation, and reference evapotranspiration emerge as the most influential predictors of stem water potential, while spectral information acts as a modulating factor that incorporates effects associated with canopy structure, vegetative vigor, and the accumulated physiological status of the crop.

The combination of PlanetScope-derived spectral indices and base band reflectance values, especially in the near-infrared region, consistently improves model performance compared with approaches based exclusively on normalized indices. The results highlight that indices related to vigor, senescence, and photosynthetic efficiency provide relevant complementary information, particularly in scenarios characterized by high spatial heterogeneity within the crop.

As a main direction for future research, future work will focus on extending the proposed methodology to Sentinel-2 imagery, as this open-access and globally available data source would substantially enhance the operational applicability of the approach. However, this transition entails additional challenges associated with both the coarser spatial resolution (10 × 10 m) and the revisit frequency (approximately 5 days). Future studies should assess the impact of spatial aggregation on the ability to detect within-field variability, as well as develop temporal integration strategies to reconstruct daily water status dynamics from less frequent satellite observations.

Additionally, it will be necessary to analyze the consistency and transferability of spectral indices and bands between PlanetScope and Sentinel-2, taking into account differences in spectral configuration and radiometric response. Validation of the approach under multi-site and multi-year conditions will contribute to the development of more robust and generalizable models, capable of supporting water stress monitoring and irrigation decision-making at broader spatial scales through the use of freely available satellite data. Given the moderate-to-strong correlation observed among several meteorological predictors (Figure 13), future work will also explore collinearity-aware feature attribution methods (e.g., correlation- or interaction-adjusted variable importance) to obtain a more fine-grained interpretation of the individual contribution of physically related atmospheric drivers, complementing the operational prediction focus of the present study.

## Figures and Tables

**Figure 1:**
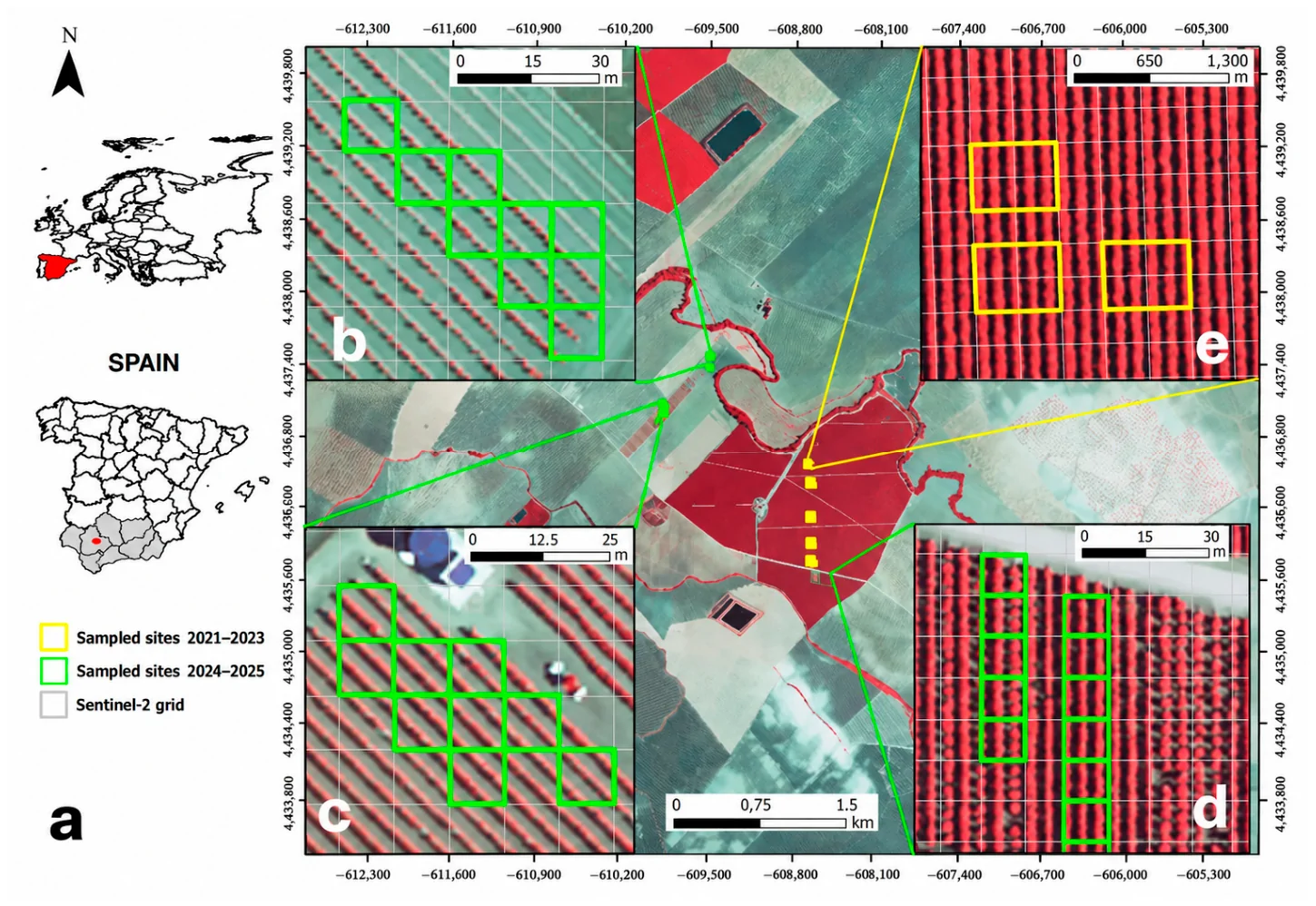
Location and spatial distribution of the sampling zones: (**a**) General location of the study area; (**b**–**d**) 2024–2025 sampling zones (10 × 10 m); (**e**) 2021–2022–2023 sampling zones (20 × 20 m).

**Figure 2:**
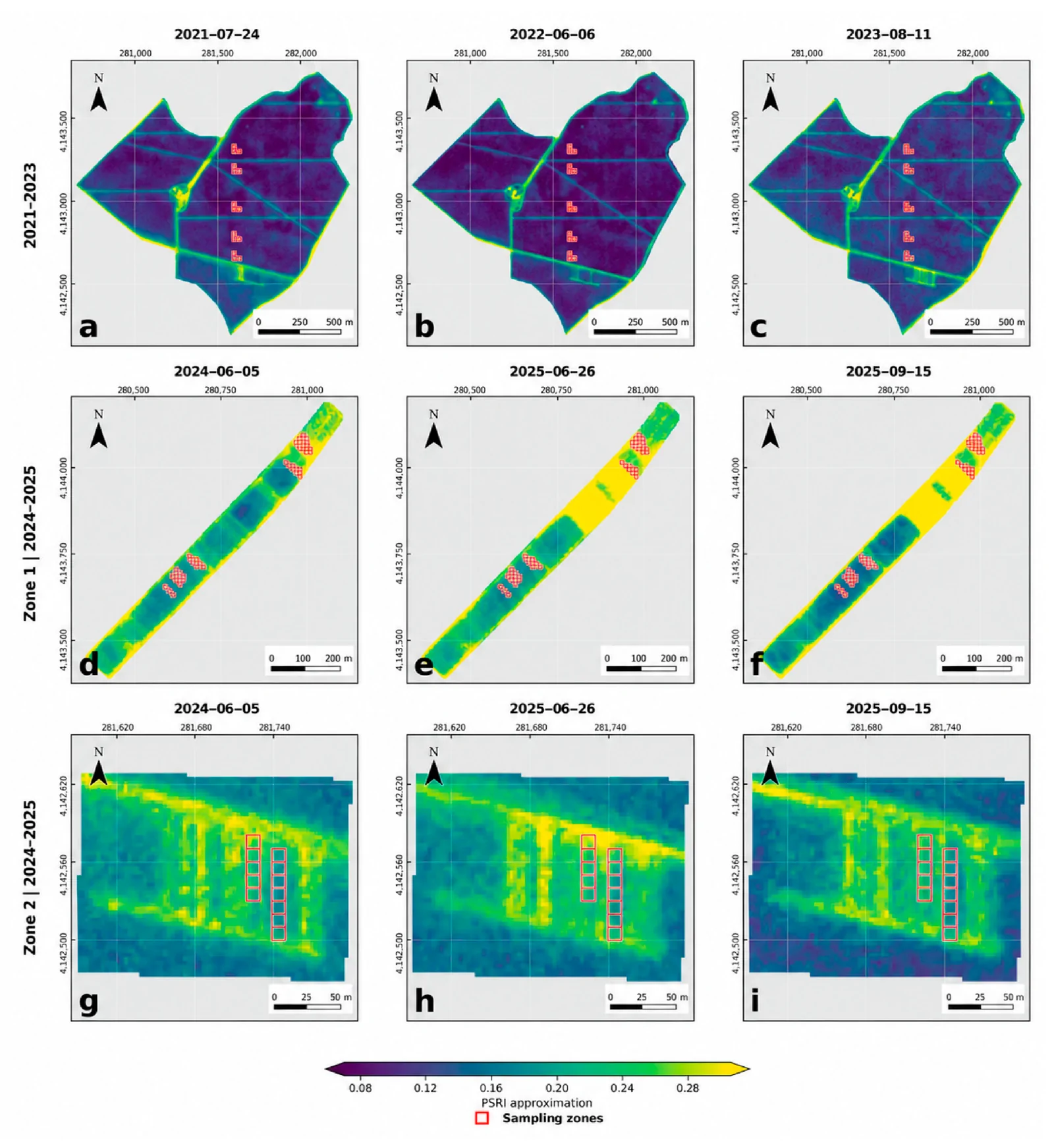
Spatial distribution of the SuperDove-based PSRI approximation for the area monitored in 2021–2023 (**a**–**c**), Zone 1 (**d**–**f**) and Zone 2 (**g**–**i**) monitored in 2024–2025. Red outlines indicate sampling zones. All panels share the same color scale; lower values are consistent with a greater contribution of green vegetation within the pixel.

**Figure 3:**
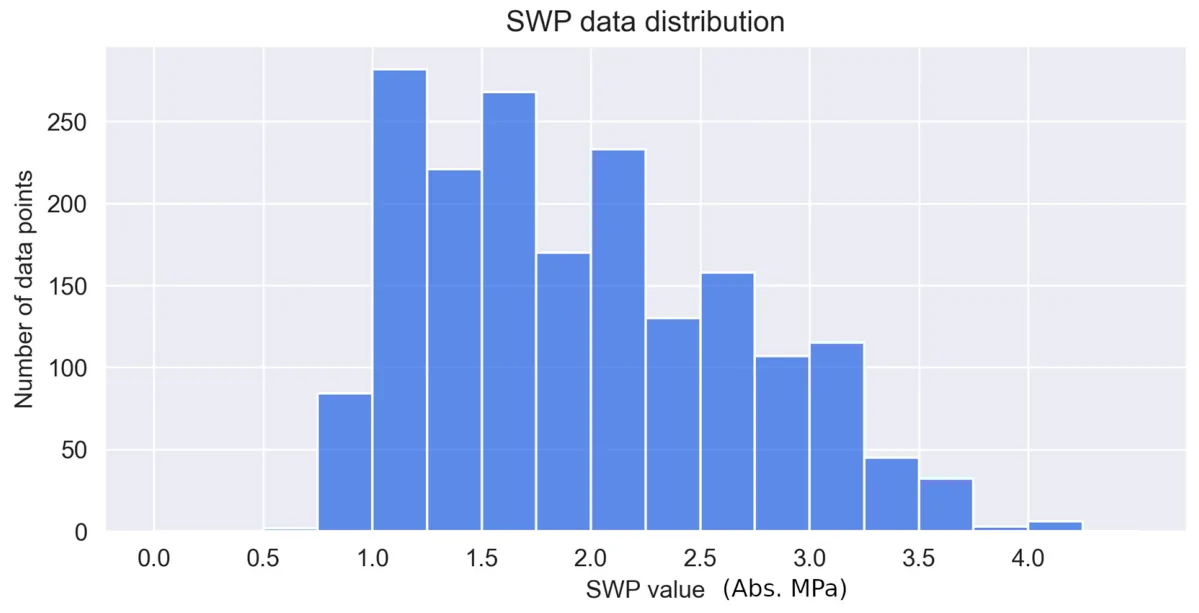
Distribution of SWP values in the dataset.

**Figure 4:**
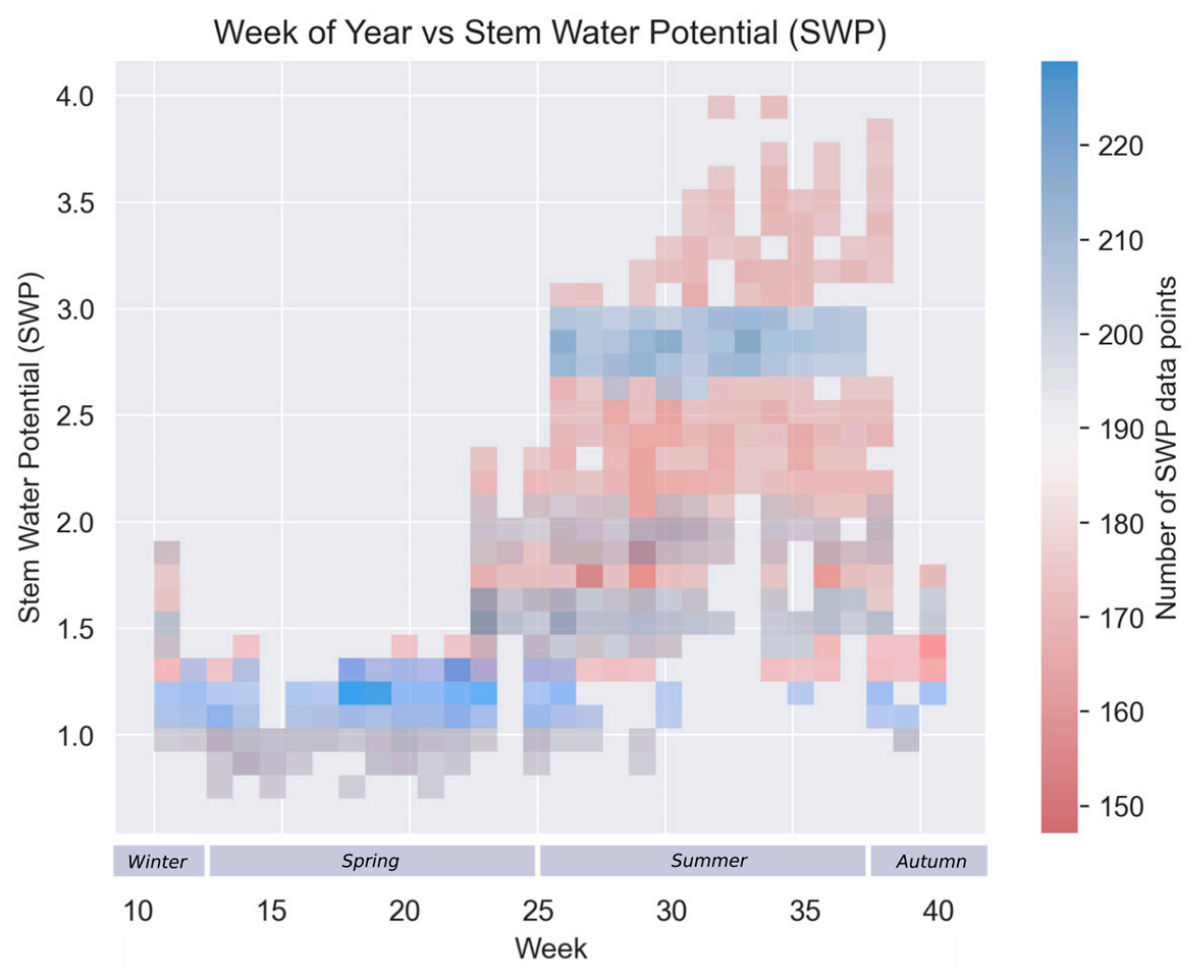
Distribution of SWP measurement datapoints as a function of the week of the year.

**Figure 5:**
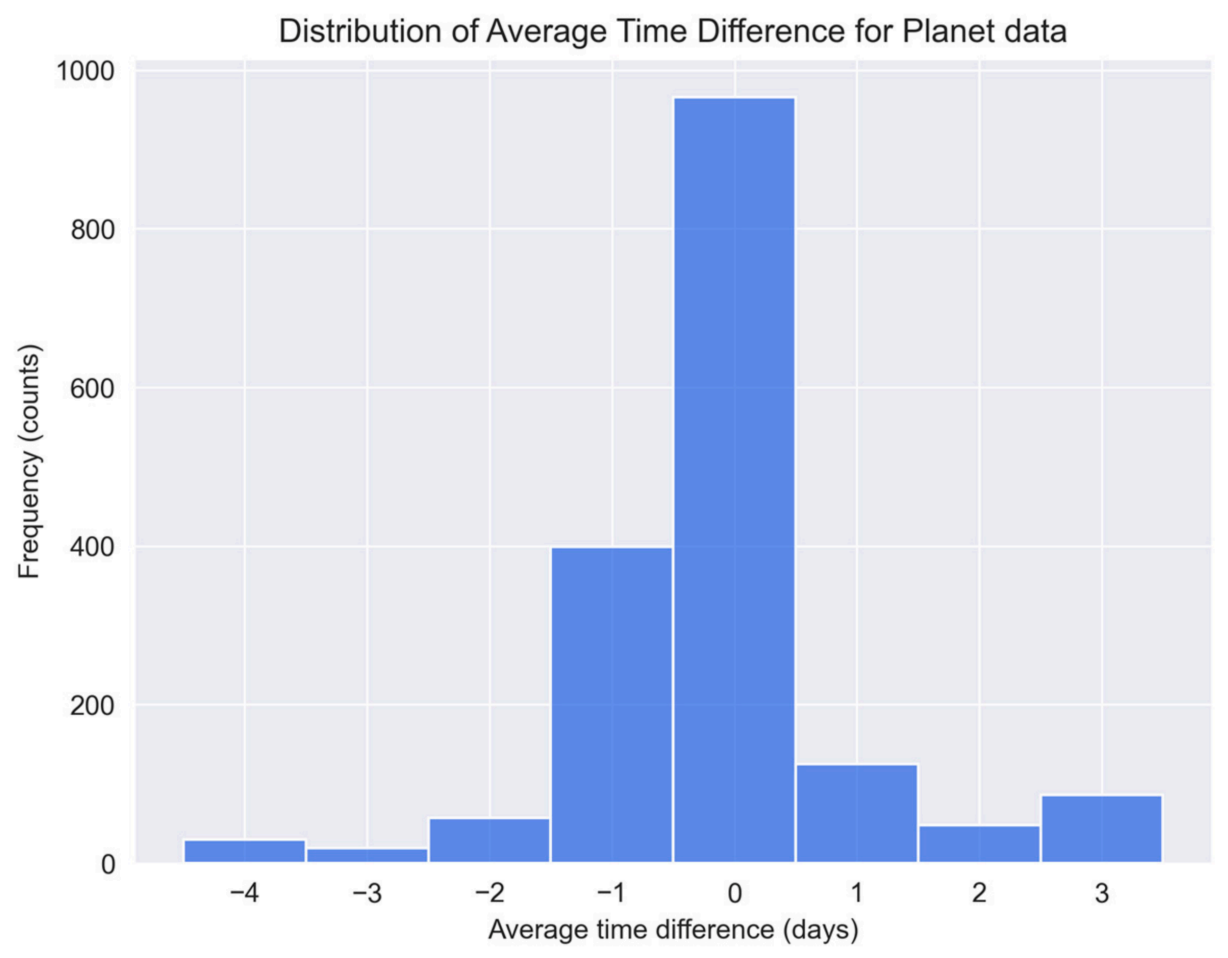
Distribution of the curated dataset (n = 1628) as a function of the temporal lag between the PlanetScope observation and the SWP measurement. Temporal differences were grouped into 1-day intervals; a negative lag indicates that the PlanetScope acquisition precedes the SWP measurement, while a positive lag indicates that it follows it.

**Figure 6:**
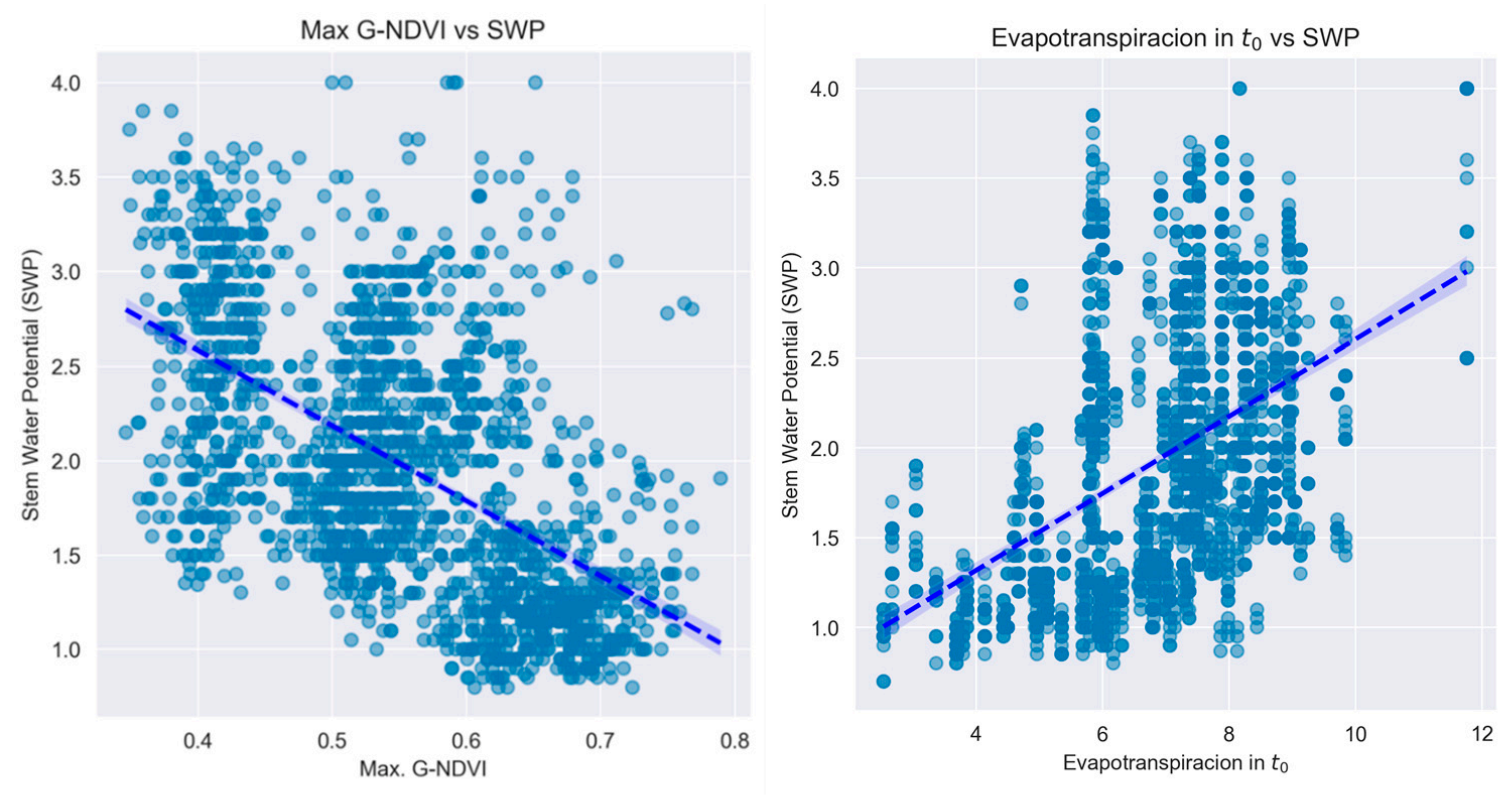
Correlation between SWP values and maximum G-NDVI index (**left**) and evapotranspiration with lag 0 (**right**).

**Figure 7:**
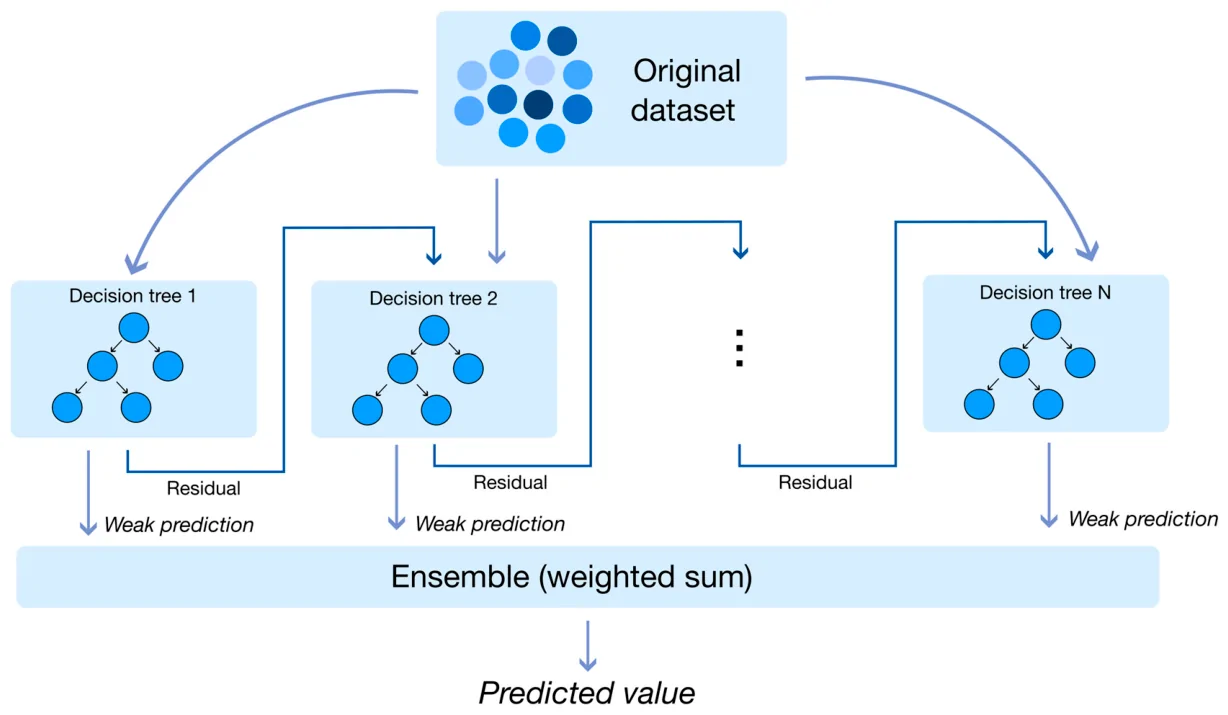
Gradient boosting architecture of XGBoost. Decision trees are trained sequentially, each fitted on the residual errors of the previous ones, and their weak predictions are combined through a weighted sum to produce the final predicted value.

**Figure 8:**
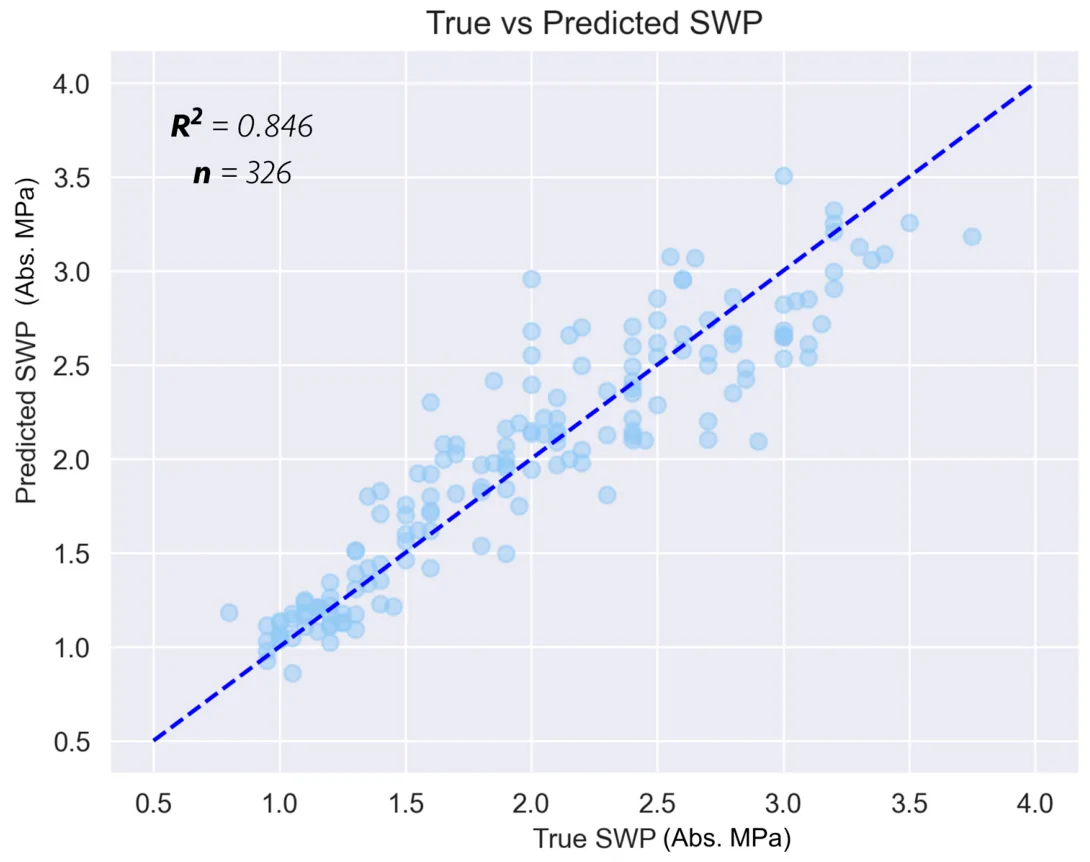
Predictions versus observed SWP values for the test set using the optimized parameter configuration.

**Figure 9:**
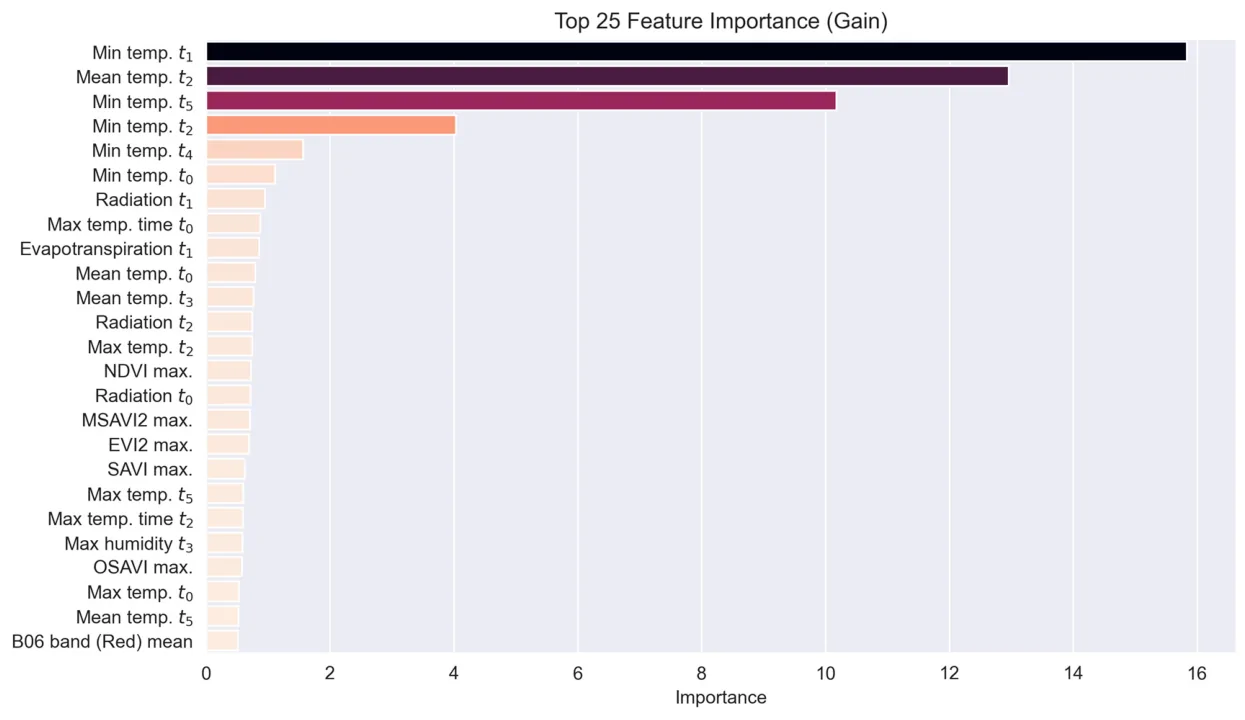
Top-25 feature importance ranked by Gain after XGBoost training.

**Figure 10:**
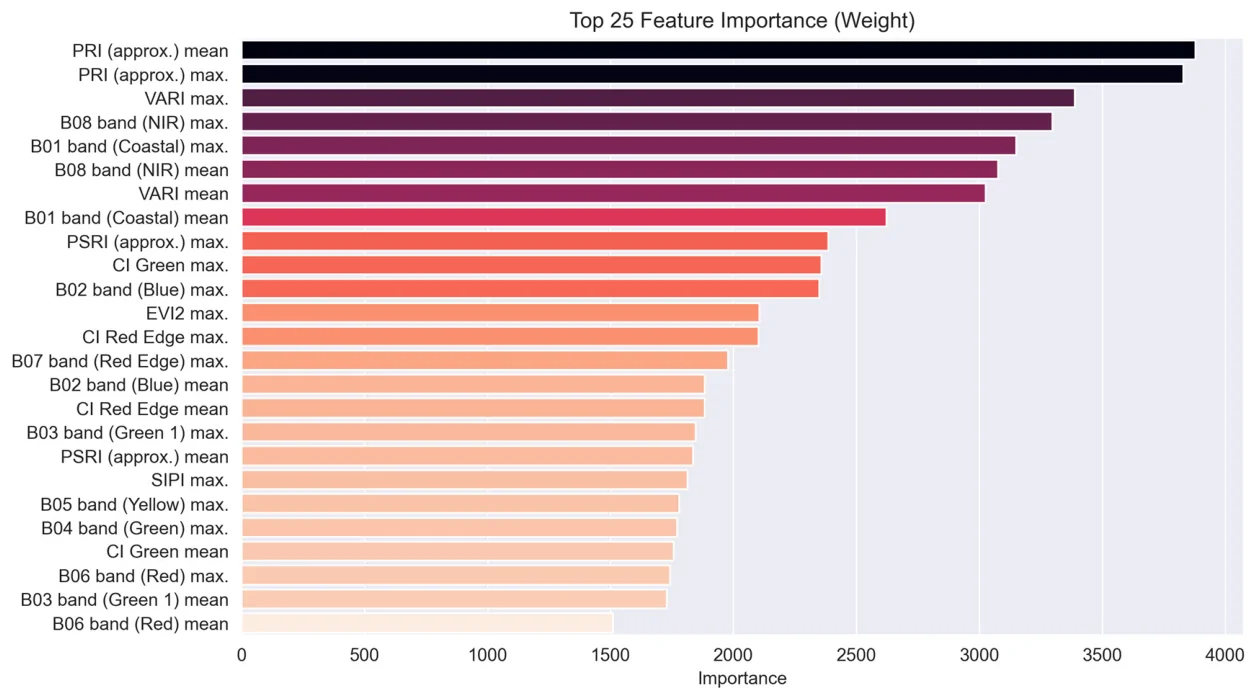
Top-25 Feature Weight importance after training with XGBoost.

**Figure 11:**
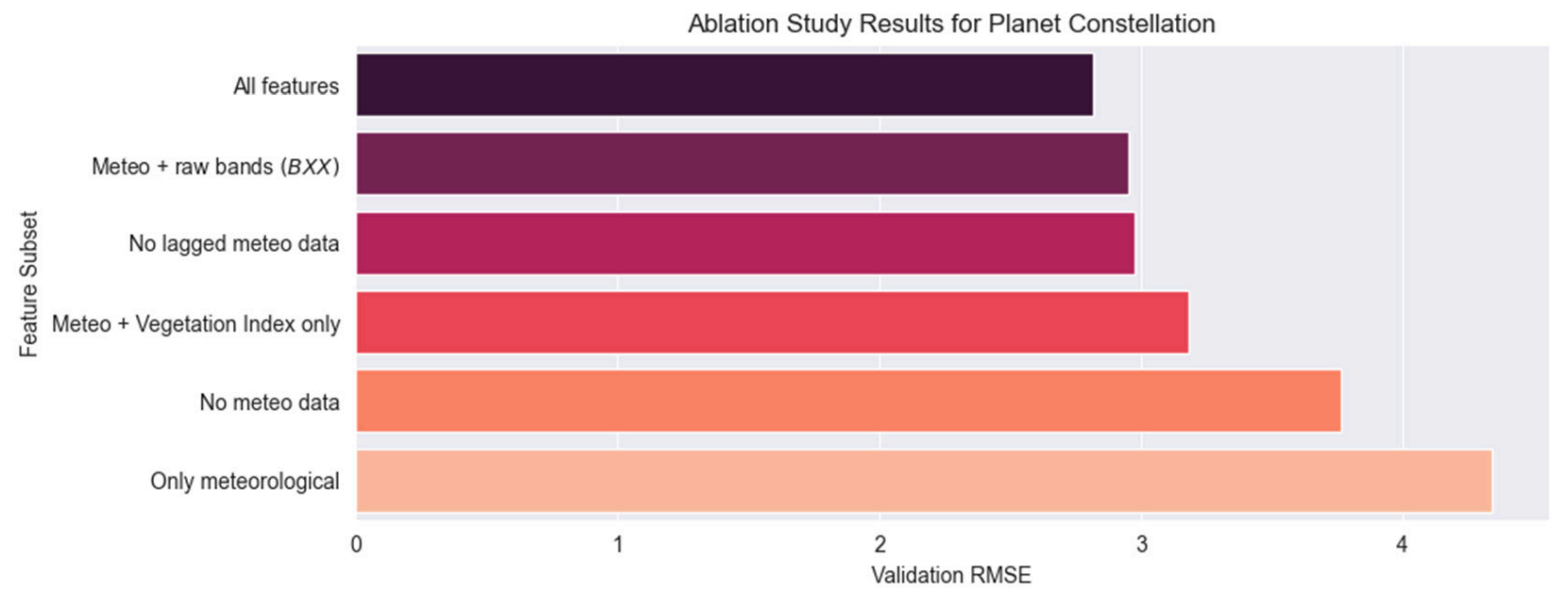
Resulting RMSE from the ablation study for the different feature groups evaluated on the test dataset.

**Figure 12:**
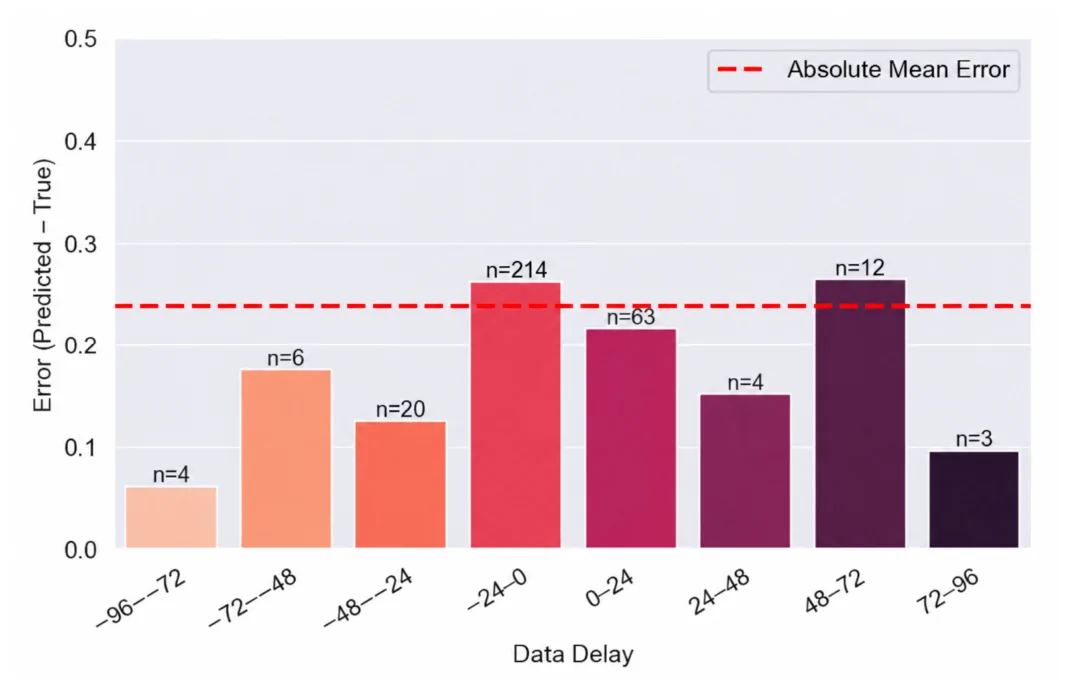
Mean prediction error on the validation set as a function of the temporal lag (in 24 h bins) between the SWP measurement and its matched PlanetScope acquisition.

**Figure 13:**
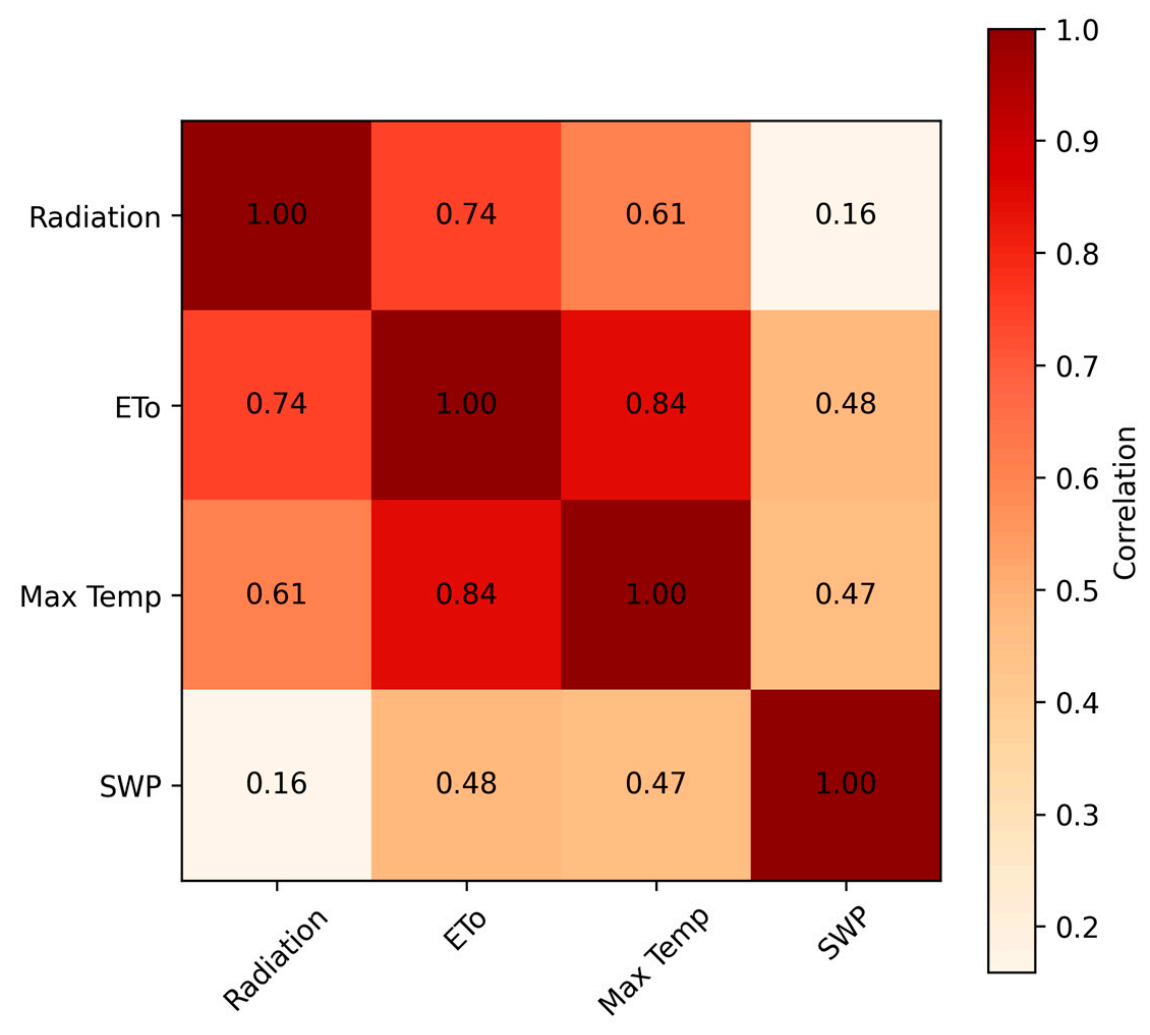
Pearson correlation matrix for same-day (t0) global solar radiation, reference evapotranspiration (ETo), and maximum air temperature, together with their correlation to SWP.

**Table 1:** Vegetation indices used in the study and their adaptation to PlanetScope spectral bands.

Vegetation Index	Formula	Description	Reference
Normalized Difference Vegetation Index (NDVI)	$NDVI=\frac{\left(NIR-R\right)}{\left(NIR+R\right)}$	Measures photosynthetically active biomass. Sensitive to soil brightness and atmospheric effects.	[53]
Soil Adjusted Vegetation Index (SAVI)	$SAVI=\frac{\left(NIR-R\right)}{\left(NIR+R+L\right)}\left(1+L\right)$	Features a soil adjustment factor to minimize background noise in sparse vegetation environments.	[54]
Optimized Soil Adjusted Vegetation Index (OSAVI)	$OSAVI=\frac{\left(NIR-R\right)}{\left(NIR+R+0.16\right)}$	Monitors regions characterized by sparse vegetation.	[55]
Modified Soil Adjusted Vegetation Index (MSAVI)	$MSAVI=\frac{\left(2NIR+1\right)-\sqrt{{\left(2NIR+1\right)}^{2}-8\left(NIR-R\right)}}{2}$	Minimizes the influence of soil background on vegetation monitoring in areas with low vegetation cover, where soil reflectance can distort data.	[56]
Chlorophyll Index—Red Edge (CI red-edge)	$CI\;red\_edge=\left(\frac{NIR}{RE}\right)-1$	Estimates leaf chlorophyll content, a key indicator of plant health, stress, and nutrient status. More sensitive to high chlorophyll content.	[57]
Chlorophyll Index— Green (CI green)	$CI\;green=\left(\frac{NIR}{G}\right)-1$	Estimates leaf chlorophyll content, a key indicator of plant health, stress, and nutrient status.	[57]
Green Normalized Difference Vegetation Index (GNDVI)	$GNDVI=\frac{\left(NIR-G\right)}{\left(NIR+G\right)}$	Estimates photosynthetic activity and chlorophyll concentration in plants. Detects plant stress or disease before they become visible to the human eye.	[58]
Normalized Difference Red Edge (NDRE)	$NDRE=\frac{\left(NIR-RE\right)}{\left(NIR+RE\right)}$	Estimates plant health and chlorophyll content. More effective for monitoring crops in mid-to-late growth stages than NDVI.	[59]
Enhanced Vegetation Index 2 (EVI2)	$EVI2=\frac{2.5(NIR-R)}{\left(NIR+2.4.R+1\right)}$	Tracks vegetation health, growth, and phenology. Better than NDVI at reducing soil noise and atmospheric effects, especially in dense areas.	[60]
Photochemical Reflectance Index (PRI) SuperDove	$PRI\_SuperDove=\frac{\left(G1-G\right)}{\left(G1+G\right)}$	Detects subtle changes in leaf pigments, especially carotenoids, linked to plant stress from light or water issues.	[61]
Photochemical Reflectance Index (PRI) Dove (approximation)	$PRI\_Dove=\frac{\left(G-B\right)}{\left(G+B\right)}$	Detects subtle changes in leaf pigments, especially carotenoids, linked to plant stress from light or water issues.	[61]
Plant Senescence Reflectance Index (PSRI)	$PSRI=\frac{\left(R-B\right)}{NIR}$	Measures the physiological stress in plants by detecting the shift in the ratio between carotenoids and chlorophyll, which occurs as a plant matures or faces severe stress.	[62]
Visible Atmospherically Resistant Index (VARI)	$VARI=\frac{\left(G-R\right)}{\left(G+R+B\right)}$	Estimates the vegetation health with RGB data, minimizing the effects of atmospheric haze and aerosols, providing more consistent data.	[63]
Structure-Insensitive Pigment Index (SIPI)	$SIPI=\frac{\left(NIR-B\right)}{\left(NIR-R\right)}$	Assess plant physiological stress by measuring the ratio of carotenoids to chlorophyll.	[64]

**Table 2:** Meteorological variables derived from the SE101_IFAPA Centro Las Torres–Tomejil agroclimatic station.

Variable	Variable Description	Calculation/Formula
Mean air temperature (°C)	Daily mean value of air temperature, representative of the general thermal conditions of the day.	$T_{\left\{mean\right\}}=\left(\frac{1}{n}\right)\sum_{i=1}^{n}T_{i}$
Maximum air temperature (°C)	Daily maximum air temperature, associated with periods of highest evaporative demand.	$T_{\left\{max\right\}}=max\left(T_{i}\right)$
Time of maximum temperature	Time of day at which the maximum air temperature is recorded.	Direct hourly record from the meteorological station.
Minimum air temperature (°C)	Daily minimum air temperature, typically occurring during nighttime or early morning hours.	$T_{\left\{min\right\}}=min\left(T_{i}\right)$
Time of minimum temperature	Time of day at which the minimum air temperature is recorded.	Direct hourly record from the meteorological station.
Mean relative humidity (%)	Daily mean relative humidity, indicative of the average atmospheric water vapor content.	$RH_{\left\{mean\right\}}=\left(\frac{1}{n}\right)\sum_{i=1}^{n}RH_{i}$
Maximum relative humidity (%)	Daily maximum relative humidity, generally associated with nocturnal or low-temperature periods.	$RH_{\left\{max\right\}}=max\left(RH_{i}\right)$
Time of maximum relative humidity	Time of day at which the maximum relative humidity is recorded.	Direct hourly record from the meteorological station.
Minimum relative humidity (%)	Daily minimum relative humidity, related to conditions of high evaporative demand.	$RH_{\left\{min\right\}}=min\left(RH_{i}\right)$
Time of minimum relative humidity	Time of day at which the minimum relative humidity is recorded.	Direct hourly record from the meteorological station.
Global solar radiation (MJ m^−2^)	Total incoming shortwave solar energy at the surface during the day, a primary driver of evapotranspiration.	$R_{g}=\sum R_{\left\{g,i\right\}}\Delta t$
Precipitation (mm)	Total rainfall accumulated during the day.	$P=\sum P_{i}$
Effective precipitation (mm)	Fraction of total precipitation effectively available to the soil–plant system.	$P_{\left\{eff\right\}}=kP$
Evapotranspiration, ET_0_ (mm)	Atmospheric evaporative demand from a well-watered reference surface.	$ET_{0}=\;FAO\;Penman-Monteit$ [68]

**Table 3:** Summary of XGBoost training hyperparameters, their meaning, and search ranges.

Hyperparameter	Meaning	Range
Num. of estimators	Number of decision trees that compose the estimator. A large number of estimators may improve accuracy, but it can also increase bias with respect to the training set.	[100, 1000]
Max. depth	It defines the maximum depth allowed for each individual tree. This parameter controls the complexity of the partitions; greater depths allow more complex relationships to be captured, but they also increase the risk of fitting noise.	[2, 20]
η	It is the learning rate parameter applied after each additive update. It determines the magnitude of each tree’s contribution to the final model. Smaller values reduce the risk of overfitting but require more iterations to achieve convergence.	[0.1, 0.5]
Subsample	It specifies the fraction of samples that are randomly selected at each iteration to train an individual tree. This parameter introduces randomness and reduces correlation among trees, thereby promoting better generalization.	[0.5, 1.0]
Colsample by Tree	It specifies the proportion of features that are randomly selected for each tree. This mechanism limits the set of predictors available at each split and helps prevent overfitting when working with high-dimensional data.	[0.5, 1.0]
Min. child weight	Minimum sum of Hessian values (or observation weights) required to create a new split. If the accumulated weight in a node does not exceed this threshold, the split is not performed. This parameter prevents partitions with few samples or low statistical support, thereby contributing to model regularization.	[1, 10]

**Table 4:** Hyperparameters resulting from the optimization using TPE parameters.

Hyperparameter	Optimized Values
Num. of estimators	619
Max. depth	17
Learning rate (η)	0.013
Subsample	0.937
Colsample by tree	0.507
Min. child weight	4.840

**Table 5:** Comparison of model performance between XGBoost training with and without hyperparameter optimization.

	TPE Optimized Parameters	Default XGBoost Library Parameters
RMSE	0.278	0.299
MAE	0.211	0.227
R^2^	0.846	0.822

**Table 6:** Resulting RMSE from the ablation study.

Ablation Subset	Features	$RMSE_{VAL}$	$R_{VAL}^{2}$	$MAE_{VAL}$	$RMSE_{CV}$	${R^{2}}_{CV}$	$MAE_{CV}$
All features	129	0.282	0.843	0.212	0.335	0.787	0.245
Meteo + raw bands	100	0.295	0.827	0.220	0.344	0.774	0.252
No lagged meteo data	59	0.298	0.824	0.227	0.346	0.772	0.253
Meteo + Vegetation Index	112	0.318	0.799	0.234	0.354	0.762	0.258
No meteo data	45	0.377	0.719	0.278	0.406	0.686	0.296

**Table 7:** Comparison of validation performance among algorithms for SWP estimation.

Algorithm	$RMSE_{VAL}$	$R_{VAL}^{2}$	$MAE_{VAL}$
XGBoost	0.27	0.84	0.21
SVR	0.30	0.81	0.23
Gaussian process	0.34	0.75	0.24
RF [37]	0.38	0.78	0.28
MLR [37]	0.49	0.58	0.40
Neural Network	0.37	0.72	0.29

**Table 8:** Comparison of cross-validation protocols assessing spatial and temporal leakage.

Validation Protocol	*RMSE*	*R* ^2^	*MAE*
Random stratified CV 80/20 split (reference)	0.33	0.78	0.24
Node (plot)-grouped holdout CV	0.294	0.837	0.218
One-season holdout CV	0.320	0.817	0.238

## Data Availability

Data is available at: https://gitlab.ratatosk.cc/syanes/estimacion-de-estres-hidrico-en-olivos/ (accessed on 21 September 2026). For any further information, please do not hesitate to contact the authors.

## Supplementary Materials

The following supporting information can be downloaded at https://www.mdpi.com/article/10.3390/s26196062/s1. Figure S1: False-color infrared composites of PlanetScope SuperDove imagery for the area monitored in 2021–2023 (a–c), and Zone 1 (d–f) and Zone 2 (g–i) monitored in 2024–2025. Near-infrared, red and green bands are displayed as red, green and blue, respectively. Yellow and green outlines indicate sampling polygons for the two monitoring periods.

## References

[1] Acutis M., Perego A., Bernardoni E., Rinaldi M. (2010). AQUATER Software as a DSS for Irrigation Management in Semi-Arid Mediterranean Areas. Ital. J. Agron..

[2] Chiarelli D.D., Nanesha H., Sardo M., Ragkos A., Rulli M.C. (2025). Water-Saving Driven Crop Reallocation Reduces Irrigation Energy Demand in the Mediterranean. Glob. Food Sec..

[3] Franco-Luesma S., Cavero J., Álvaro-Fuentes J. (2025). Relevance of the Irrigation and Soil Management System to Optimize Maize Crop Production under Semiarid Mediterranean Conditions. Agric. Water Manag..

[4] Arbizu-Milagro J., Castillo-Ruiz F.J., Tascón A., Peña J.M. (2023). Effects of Regulated, Precision and Continuous Deficit Irrigation on the Growth and Productivity of a Young Super High-Density Olive Orchard. Agric. Water Manag..

[5] Alderotti F., Lo Piccolo E., Brunetti C., Stefano G., Ugolini T., Cecchi L., Beccaluva M., Renna L., Detti C., Ferrini F. (2025). Cultivar-Specific Responses of Young Olive Trees to Water Deficit: Impacts on Physiology, Leaf Anatomy, and Fruit Quality in “Arbequina”, “Leccio Del Corno” and ‘Maurino’. Plant Physiol. Biochem..

[6] Deforce B., Baesens B., Diels J., Janssens P., Bonet Pérez de León L., Serral Asensio E. (2025). Deep Multi-Task Learning for Stem Water Potential Prediction: Actionable Guidelines. Comput. Electron. Agric..

[7] Feng X., Yan Z., Liu N., Liu Q., Shao L., Liu X. (2025). Optimizing Soil Water Sensor Placement and Irrigation Thresholds for Winter Wheat: A Data-Driven Approach to Efficient Water Management. Agric. Water Manag..

[8] Fan X., Che H., Wang Y., Chen D., Hu X. (2025). Effects of Deep Storage Irrigation on Summer Maize Yield Formation and Safety Irrigation Threshold for Maximizing Flood Resources Utilization. Agric. Water Manag..

[9] Moriana A., Pérez-López D., Prieto M.H., Ramírez-Santa-Pau M., Pérez-Rodriguez J.M. (2012). Midday Stem Water Potential as a Useful Tool for Estimating Irrigation Requirements in Olive Trees. Agric. Water Manag..

[10] Corell M., Martín-Palomo M.J., Girón I., Andreu L., Galindo A., Centeno A., Pérez-López D., Moriana A. (2020). Stem Water Potential-Based Regulated Deficit Irrigation Scheduling for Olive Table Trees. Agric. Water Manag..

[11] Sánchez-Piñero M., Corell M., Moriana A., Castro-Valdecantos P., Martin-Palomo M.J. (2023). Managing Water Stress in Olive (*Olea europaea* L.) Orchards Using Reference Equations for Midday Stem Water Potential. Horticulturae.

[12] Mortazavi M., Carpin S., Toudeshki A., Ehsani R. (2025). A Practical Data-Driven Approach for Precise Stem Water Potential Monitoring in Pistachio and Almond Orchards Using Supervised Machine Learning Algorithms. Comput. Electron. Agric..

[13] Ohana-Levi N., Zachs I., Hagag N., Shemesh L., Netzer Y. (2022). Grapevine Stem Water Potential Estimation Based on Sensor Fusion. Comput. Electron. Agric..

[14] Divya R., Chinnaiyan R. (2019). Reliable AI-Based Smart Sensors for Managing Irrigation Resources in Agriculture—A Review. Lecture Notes on Data Engineering and Communications Technologies.

[15] European Commission-H2020 project (2019). A Precise Irrigation Monitoring System to Provide an Accurate Measurement of Water Status in Crops. Eur. Comm..

[16] Kisekka I., Peddinti S.R., Savchik P., Yang L., Culumber M., Bali K., Milliron L., Edwards E., Nocco M., Reyes C.A. (2024). Multisite Evaluation of Microtensiometer and Osmotic Cell Stem Water Potential Sensors in Almond Orchards. Comput. Electron. Agric..

[17] Vaccaro G., Fusco M., Alagna V., Franco L., Motisi A., Iovino M. (2025). Assessing Microtensiometers for Monitoring Stem Water Potential in Mandarin (*Citrus reticulata* Blanco) Orchard under Different Irrigation Regimes. Agric. Water Manag..

[18] Jones H.G. (2004). Irrigation Scheduling: Advantages and Pitfalls of Plant-Based Methods. J. Exp. Bot..

[19] Vidican R., Mălinaș A., Ranta O., Moldovan C., Marian O., Ghețe A., Ghișe C.R., Popovici F., Cătunescu G.M. (2023). Using Remote Sensing Vegetation Indices for the Discrimination and Monitoring of Agricultural Crops: A Critical Review. Agronomy.

[20] Skendžić S., Zovko M., Lešić V., Pajač Živković I., Lemić D. (2023). Detection and Evaluation of Environmental Stress in Winter Wheat Using Remote and Proximal Sensing Methods and Vegetation Indices—A Review. Diversity.

[21] El-baki M.S.A., Ibrahim M.M., Elsayed S., Yaseen Z.M., El-Fattah N.G.A. (2025). Water Status and Plant Traits of Dry Bean Assessment Using Integrated Spectral Reflectance and RGB Image Indices with Artificial Intelligence. Sci. Rep..

[22] Dong H., Dong J., Sun S., Bai T., Zhao D., Yin Y., Shen X., Wang Y., Zhang Z., Wang Y. (2024). Crop Water Stress Detection Based on UAV Remote Sensing Systems. Agric. Water Manag..

[23] Zhao J., Feng H., Xu T., Xiao J., Guerrieri R., Liu S., Wu X., He X., He X. (2021). Physiological and Environmental Control on Ecosystem Water Use Efficiency in Response to Drought across the Northern Hemisphere. Sci. Total Environ..

[24] Sadok W., Lopez J.R., Smith K.P. (2021). Transpiration Increases under High-Temperature Stress: Potential Mechanisms, Trade-Offs and Prospects for Crop Resilience in a Warming World. Plant Cell Environ..

[25] Ryckewaert M., Héran D., Simonneau T., Abdelghafour F., Boulord R., Saurin N., Moura D., Mas-Garcia S., Bendoula R. (2022). Physiological Variable Predictions Using VIS–NIR Spectroscopy for Water Stress Detection on Grapevine: Interest in Combining Climate Data Using Multiblock Method. Comput. Electron. Agric..

[26] Shi B., Yuan Y., Zhuang T., Xu X., Schmidhalter U., Ata-UI-Karim S.T., Zhao B., Liu X., Tian Y., Zhu Y. (2022). Improving Water Status Prediction of Winter Wheat Using Multi-Source Data with Machine Learning. Eur. J. Agron..

[27] Ahmad U., Alvino A., Marino S. (2021). A Review of Crop Water Stress Assessment Using Remote Sensing. Remote Sens..

[28] Verrelst J., Malenovský Z., Van der Tol C., Camps-Valls G., Gastellu-Etchegorry J.P., Lewis P., North P., Moreno J. (2019). Quantifying Vegetation Biophysical Variables from Imaging Spectroscopy Data: A Review on Retrieval Methods. Surv. Geophys..

[29] He X., Li Y., Liu S., Xu T., Chen F., Li Z., Zhang Z., Liu R., Song L., Xu Z. (2023). Improving Regional Climate Simulations Based on a Hybrid Data Assimilation and Machine Learning Method. Hydrol. Earth Syst. Sci..

[30] Fu Y., Zhu Z., Liu L., Zhan W., He T., Shen H., Zhao J., Liu Y., Zhang H., Liu Z. (2024). Remote Sensing Time Series Analysis: A Review of Data and Applications. J. Remote Sens..

[31] Kamm S., Veekati S.S., Müller T., Jazdi N., Weyrich M. (2023). A Survey on Machine Learning Based Analysis of Heterogeneous Data in Industrial Automation. Comput. Ind..

[32] Rahaman M., Southworth J., Amanambu A.C., Tefera B.B., Alruzuq A.R., Safaei M., Hasan M.M., Smith A.C. (2025). Combining Deep Learning and Machine Learning Techniques to Track Air Pollution in Relation to Vegetation Cover Utilizing Remotely Sensed Data. J. Environ. Manag..

[33] Hangloo S., Arora B. (2025). Multimodal Fusion Techniques: Review, Data Representation, Information Fusion, and Application Areas. Neurocomputing.

[34] King B.A., Shellie K.C. (2023). A Crop Water Stress Index Based Internet of Things Decision Support System for Precision Irrigation of Wine Grape. Smart Agric. Technol..

[35] Bwambale E., Abagale F.K., Anornu G.K. (2022). Smart Irrigation Monitoring and Control Strategies for Improving Water Use Efficiency in Precision Agriculture: A Review. Agric. Water Manag..

[36] Kamarudin M.H., Ismail Z.H., Saidi N.B. (2021). Deep Learning Sensor Fusion in Plant Water Stress Assessment: A Comprehensive Review. Appl. Sci..

[37] Garofalo S.P., Giannico V., Costanza L., Alhajj Ali S., Camposeo S., Lopriore G., Pedrero Salcedo F., Vivaldi G.A. (2024). Prediction of Stem Water Potential in Olive Orchards Using High-Resolution Planet Satellite Images and Machine Learning Techniques. Agronomy.

[38] Savchik P., Nocco M., Kisekka I. (2025). Mapping Almond Stem Water Potential Using Machine Learning and Multispectral Imagery. Irrig. Sci..

[39] Carrasco-Benavides M., Gonzalez Viejo C., Tongson E., Baffico-Hernández A., Ávila-Sánchez C., Mora M., Fuentes S. (2022). Water Status Estimation of Cherry Trees Using Infrared Thermal Imagery Coupled with Supervised Machine Learning Modeling. Comput. Electron. Agric..

[40] Zambrano F., Herrera A., Olguín M., Miranda M., Garrido J., Almeida A.M. (2025). Prediction of the Daily Spatial Variation of Stem Water Potential in Cherry Orchards Using Weather and Sentinel-2 Data. Agric. Water Manag..

[41] Chen T., Guestrin C. A Scalable Tree Boosting System. Proceedings of the 22nd ACM SIGKDD International Conference on Knowledge Discovery and Data Mining.

[42] Rural Innovation Hub Rural Innovation Hub–Centro de Innovación Rural en Finca El Valenciano Innovación, Promoción del Emprendimiento y Transferencia de Conocimiento en un Entorno Único. https://ruralinnovationhub.com/.

[43] Deuser Tech Group CENTAR-IA|Centro de Telecontrol Inteligente Para Multi-Asistencia Del Colectivo Agrario (CIA-112). https://www.deuser.es/proyectos/centar-ia-centro-de-telecontrol-inteligente-para-multi-asistencia-del-colectivo-agrario-cia-112/.

[44] Sanz-Cortés F., Martinez-Calvo J., Badenes M.L., Bleiholder H., Hack H., Llacer G., Meier U. (2002). Phenological Growth Stages of Olive Trees (*Olea europaea*). Ann. Appl. Biol..

[45] IVIA Technical Data Sheet. http://riegos.ivia.es/ficheros/recursos/ficha_tecnica_camara_de_presion.pdf.

[46] Fulton A., Buchner R., Olson B., Schwankl L., Gilles C., Bertagna N., Walton J., Shackel K. (2001). Rapid Equilibration of Leaf and Stem Water Potential under Field Conditions in Almonds, Walnuts, and Prunes. Horttechnology.

[47] Planet (2025). PlanetScope Product Specifications PLANET.COM.

[48] Planet Education and Research Program. https://www.planet.com/industries/education-and-research/.

[49] Planet PlanetScope. https://docs.planet.com/data/imagery/planetscope/.

[50] Chen Q., Vaudour E., Richer-de-Forges A.C., Arrouays D. (2025). Spectral Indices in Remote Sensing of Soil: Definition, Popularity, and Issues. A Critical Overview. Remote Sens. Environ..

[51] Qi Y., Lou M., Liu Y., Li L., Yang Z., Nie W. (2026). Advancing Image Super-Resolution Techniques in Remote Sensing: A Comprehensive Survey. ISPRS J. Photogramm. Remote Sens..

[52] Radočaj D., Šiljeg A., Marinović R., Jurišić M. (2023). State of Major Vegetation Indices in Precision Agriculture Studies Indexed in Web of Science: A Review. Agriculture.

[53] Rouse J.W., Haas R.H., Schell J.A., Deering D.W. (1974). Monitoring Vegetation Systems in the Great Plains with ERTS-NASA Technical Reports Server (NTRS). NTRS-NASA Tech. Rep. Serv..

[54] Huete A.R. (1988). A Soil-Adjusted Vegetation Index (SAVI). Remote Sens. Environ..

[55] Rondeaux G., Steven M., Baret F. (1996). Optimization of Soil-Adjusted Vegetation Indices. Remote Sens. Environ..

[56] Qi J., Chehbouni A., Huete A.R., Kerr Y.H., Sorooshian S. (1994). A Modified Soil Adjusted Vegetation Index. Remote Sens. Environ..

[57] Gitelson A.A., Viña A., Ciganda V., Rundquist D.C., Arkebauer T.J. (2005). Remote Estimation of Canopy Chlorophyll Content in Crops. Geophys. Res. Lett..

[58] Gitelson A.A., Kaufman Y.J., Merzlyak M.N. (1996). Use of a Green Channel in Remote Sensing of Global Vegetation from EOS- MODIS. Remote Sens. Environ..

[59] Gitelson A., Merzlyak M.N. (1994). Spectral Reflectance Changes Associated with Autumn Senescence of *Aesculus hippocastanum* L. and *Acer platanoides* L. Leaves. Spectral Features and Relation to Chlorophyll Estimation. J. Plant Physiol..

[60] Jiang Z., Huete A.R., Didan K., Miura T. (2008). Development of a Two-Band Enhanced Vegetation Index without a Blue Band. Remote Sens. Environ..

[61] Gamon J.A., Serrano L., Surfus J.S. (1997). The Photochemical Reflectance Index: An Optical Indicator of Photosynthetic Radiation Use Efficiency across Species, Functional Types, and Nutrient Levels. Oecologia.

[62] Merzlyak M.N., Gitelson A.A., Chivkunova O.B., Rakitin V.Y. (1999). Non-Destructive Optical Detection of Pigment Changes during Leaf Senescence and Fruit Ripening. Physiol. Plant..

[63] Gitelson A.A., Kaufman Y.J., Stark R., Rundquist D. (2002). Novel Algorithms for Remote Estimation of Vegetation Fraction. Remote Sens. Environ..

[64] Penuelas J., Baret F., Filella I. (1995). Semi-Empirical Indices to Assess Carotenoids/Chlorophyll a Ratio from Leaf Spectral Reflectance. Photosynthetica.

[65] Li W., Feng X., Du T., Kang S., Zhang H., Tong L. (2026). Estimating Maize Canopy Water Content Using UAV-Based Multispectral–Thermal Infrared Imagery and Canopy Signal Distributional Features. Front. Plant Sci..

[66] IFAPA Datos de La Estación | Instituto de Investigación y Formación Agraria y Pesquera. https://www.juntadeandalucia.es/agriculturaypesca/ifapa/riaweb/web/estacion/41/101.

[67] Ministry for Digital Transformation and the Civil Service The Agroclimatic Information System for Irrigation (SiAR)|Datos.Gob.Es. https://datos.gob.es/en/blog/agroclimatic-information-system-irrigation-siar.

[68] Pereira L.S., Allen R.G., Smith M., Raes D. (2015). Crop Evapotranspiration Estimation with FAO56: Past and Future. Agric. Water Manag..

[69] Lima Marinho T., do Nascimento D.C., Pimentel B.A. (2024). Optimization on Selecting XGBoost Hyperparameters Using Meta-Learning. Expert Syst..

[70] Ozaki Y., Tanigaki Y., Watanabe S., Onishi M. Multiobjective Tree-Structured Parzen Estimator for Computationally Expensive Optimization Problems. Proceedings of the 2020 Genetic and Evolutionary Computation Conference.

[71] Mirás-Avalos J.M., Escalona J.M., Pérez-Álvarez E.P., Romero P., Botia P., Navarro J., Torres N., Santesteban L.G., Uriarte D., Intrigliolo D.S. (2024). Upgrading and Validating a Soil Water Balance Model to Predict Stem Water Potential in Vineyards. Agric. For. Meteorol..

