# Supplementary material for: Machine Learning-Based Prediction of Stem Water Potential in Olive Orchards Using PlanetScope Imagery and Meteorological Data
Source: Sensors (Basel). 2026 Sep 24;26(19):6062. doi: 10.3390/s26196062 (PMC13667413; doi:10.3390/s26196062)
Supplement: Supplementary file 1 [file sensors-26-06062-s001.zip › sensors-4502414-supplementary.pdf]

Supplementary Material S1. PlanetScope SuperDove imagery across acquisition periods

This supplementary material illustrates the spatial and temporal variation in PlanetScope SuperDove imagery through false-color infrared composites of selected acquisitions spanning 2021–2025. It includes the study area monitored during 2021–2023 and the two zones monitored during 2024–2025. Acquisition dates were selected to represent different periods within the available dataset. Identical dates are shown for both zones monitored during 2024–2025 to facilitate comparison.

The composites use orthorectified surface reflectance imagery with a product pixel size of 3 m. Near-infrared, red and green bands are assigned to the red, green and blue display channels, respectively, so vegetation with strong near-infrared reflectance appears red. A common contrast stretch and gamma adjustment are applied across all panels to facilitate visual comparison. However, these composites are intended for qualitative interpretation: apparent differences in color or brightness should not be interpreted as proportional changes in vegetation vigor, as they may also reflect differences in soil background, canopy cover and acquisition conditions. Sampling polygons and enlarged views provide spatial context for the observations.

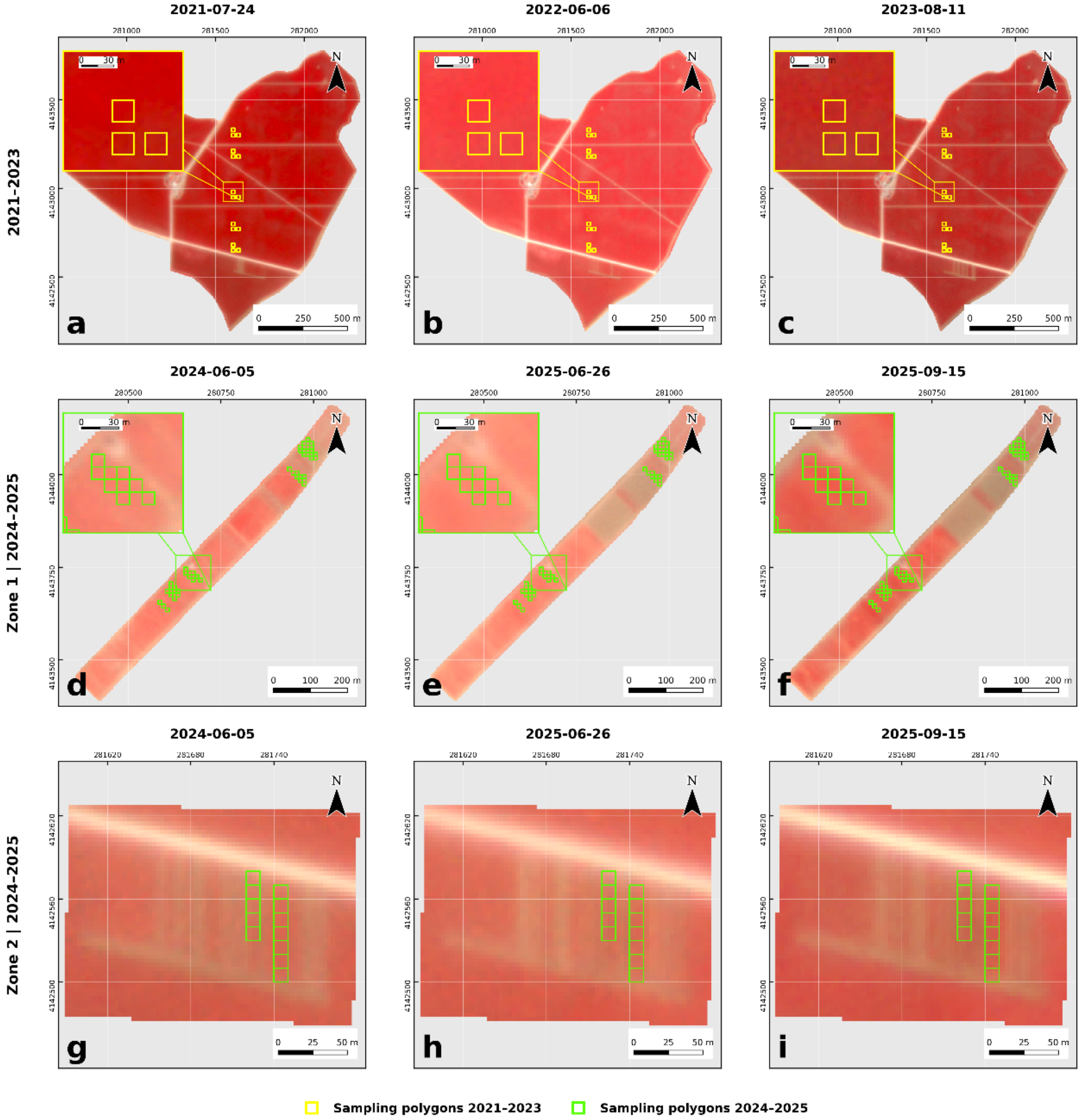

Figure S1. False-color infrared composites of PlanetScope SuperDove imagery for the area monitored in 2021–2023 (a–c), and Zone 1 (d–f) and Zone 2 (g–i) monitored in 2024–2025. Near-infrared, red and green bands are displayed as red, green and blue, respectively. Yellow and green outlines indicate sampling polygons for the two monitoring periods.
